# Birth weight and school absences and attainment: a longitudinal linked cohort study of compulsory schooling in England

**DOI:** 10.1136/archdischild-2025-328611

**Published:** 2025-04-25

**Authors:** Gergő Baranyi, Katie Harron, Emla Fitzsimons

**Affiliations:** 1 Centre for Longitudinal Studies, UCL Social Research Institute, University College London, London, UK; 2 Population, Policy & Practice Department, UCL Great Ormond Street Institute of Child Health, University College London, London, UK

**Keywords:** Child Health, Epidemiology, Infant Development

## Abstract

**Objective:**

To explore how birth weight and size-for-gestation may contribute to school absences and educational attainment and whether there are different associations across sex and income groups.

**Design:**

Longitudinal linked cohort study.

**Methods:**

Data were drawn from the Millennium Cohort Study, a nationally representative cohort of children born in 2000–2001; percentage of authorised and unauthorised absences from Year 1 to Year 11, and Key Stage test scores at ages 7, 11 and 16 in English and Maths were linked from the National Pupil Database. Birth outcomes and covariates were derived from the 9-month survey, and linear regressions with complex survey weights were fitted.

**Results:**

Being born small-for-gestational-age (vs average-for-gestational-age) was associated with an increase of 0.47%, 0.55% and 0.40% in authorised absences in Years 1, 3 and 4 (n=6659) and with a reduction of 0.16–0.26 SD in all English and Maths test scores (n=6204). Similar associations were found for birth weight. After adjusting for prior test scores, English (b=0.07) and Maths (b=0.05) performance at age 11 remained associated with birth weight. Socioeconomic status modified the associations: there were larger disparities in test scores among higher-income families, suggesting that higher income did not compensate for being born small-for-gestational-age.

**Conclusion:**

Children born smaller missed slightly more classes (~1 day per year) during primary school and had lower English and Maths performance across compulsory education. Exploring specific health conditions and understanding how education and health systems can work together to support children may help to reduce the burden.

**WHAT IS ALREADY KNOWN ON THIS TOPIC**

- Birth weight is a key determinant of life course development and health.
- Less is known about how birth weight is associated with educational outcomes, especially with school absences and test scores.

**WHAT THIS STUDY ADDS**

- Children born with low birth weight and small-for-gestational-age had more authorised absences during primary school and lower English and Maths test scores.
- Higher household income did not compensate for being born small-for-gestational-age.

**HOW THIS STUDY MIGHT AFFECT RESEARCH, PRACTICE OR POLICY**

- Research should identify specific health conditions related to school absences and performance. Joined support provided by educational and health systems might reduce the burden of low birth weight on educational outcomes.

## Introduction

 In 2020, one in seven newborns had low birth weight (<2500 g) globally.^1^ Although corresponding figures are lower in high-income Western economies,^1^ data from the Office for National Statistics suggests that in 2019, still 6.9% of newborns in England and Wales were affected.^2^ Low birth weight (BW) is a global index of poor in utero development, affected by both length of gestation and intrauterine growth, and is a key determinant of healthy life course development linked not only to health^3^ and mortality^4^ but also to earnings.^5^ Given the percentage of low BW newborns has been fairly stable in England since 2010,^2^ identifying risk factors, reducing the disease burden^6^ and addressing the needs of this vulnerable population remains a public health priority.

Birth outcomes are also associated with school attainment. Infants born preterm (<37 weeks) are less likely to meet expected levels of attainment^7 8^ and tend to receive lower grades than their term-born peers.^9^ Although fewer studies have focused on BW, they found a link to attainment^10^ and performance,^11^ suggesting that intrauterine growth restriction (in addition to preterm birth) might also contribute to educational outcomes.^12^ School absence plays a crucial part in educational attainment^13^ and labour market outcomes.^14^ In recent years, consistently high rates of absences became a significant policy concern^15^; still, it remains unclear how BW is linked to school attendance. BW might impact school outcomes differently among boys and girls,^10^ and parents with better resources might be able to compensate for the negative effects of low BW.^9^ Still, studies on effect modification are limited and conflicting; understanding differential associations by sex and socioeconomic status requires more attention and could help to build better-targeted interventions.

Linking educational records to a nationally representative birth cohort, we assessed how BW and size-for-gestation were associated with authorised and unauthorised school absences as well as English and Maths test scores across the 11 years of compulsory schooling in England. Additionally, we explored how sex and household income modified these associations.

## Methods

The Millennium Cohort Study (MCS) is a nationally representative cohort of approximately 19 000 children born in 2000/2001 in the UK, with oversampling in disadvantaged areas and ethnic minorities. Children were randomly selected around 9 months of age (ie, Sweep 1), with follow-ups coinciding with key developmental stages.^16^ During Sweep 4 (age 7), participants’ carers were asked to provide informed written consent to link pupils’ educational records up to age 16.^17^ For consenting participants living in England, educational records were extracted from the National Pupil Database by the Department of Education^18^; the overall successful linkage rate was 99.4%.^17^ MCS participants residing in England during Sweep 4 were included in the final sample if they were from singleton births, joined MCS in Sweep 1 (692 families joined the study in Sweep 2, but gestational age was not available for them^16^) and were linked to educational records.

### Birth weight

Information on BW and gestational age was provided by the main carers. We replaced implausible BWs (ie, outside of 4 SD of the population mean for gestational age) as missing^19^ and excluded them from the analyses (0.9%). In addition to the cleaned continuous BW variable expressed in kilograms, we computed a dichotomous marker for size-for-gestation, identifying small-for-gestational-age (SGA) participants where BW was below the 10th centile of the gestational age- and sex-specific reference value using the INTERGROWTH-21st standards.^20^

### Educational records

The National Pupil Dataset captures a wide range of administrative data on English children studying in state-funded schools, providing a near whole population coverage.^21^

#### Attendance between Year 1 and Year 11

The annual percentage of sessions missed due to authorised (ie, permitted by school representative) and unauthorised (ie, unjustified, unpermitted and arrival after registration closed) absence was computed using the total number of sessions possible and the number of sessions missed during each academic year; there are two sessions (morning and afternoon) per day.^22^ Academic years consist of three terms, with each separated into two half terms. Prior to the academic year 2012/2013, the Department for Education only collected attendance for the first five half terms,^22^ and so we used these variables across the total study period.

#### Key Stages (KS) 1, 2 and 4 attainments

Test scores for English and Maths were used to measure school attainment. The national curriculum in England is divided into four KS: KS1 Year 1–2 (ages 5–7), KS2 Year 3–6 (ages 7–11), KS3 Year 7–9 (ages 11–14) and KS4 Year 10–11 (ages 14–16).^23^ Teacher-assessed English reading and Maths tests from KS1 (age 7), national test marks for English and Maths from KS2 (age 11), English and Maths General Certificate of Secondary Education (GCSE) scores from KS4 (age 16) were used; we also included Attainment 8 (A8) scores (performance across eight GCSE-level qualifications, with English and Maths double-weighted) from KS4. Scores were standardised to support the comparison of effect sizes across different test scores. KS tests taking place in academic years other than 2007/2008 (KS1), 2011/2012 (KS2) and 2016/2017 (KS4) were excluded from analyses (0.3%–0.7%) to support alignment between absence and attainment data.

### Covariates

Confounders were identified using a directed acyclic graph (online supplemental figure 1) and derived from Sweep 1 (if missing Sweep 2). They included sex (male and female), month of birth, ethnic group (Black or Black British, Indian, Pakistani and Bangladeshi, White, Mixed and Other ethnic groups), maternal age at birth (in years), maternal smoking (never, reduced/stopped before pregnancy, smoked during pregnancy and smoked at other times), main caregiver’s partnership status (single parent and living with partner), number of siblings (none, one and two or more). In addition to individual-level factors, we also considered the highest household education, household income quintiles and household tenure. The highest household education (main caregiver or their partner, whichever is higher) is indicated by the National Vocational Qualification (NVQ) scale (none/unknown, overseas only, NVQ1, NVQ2, NVQ3, NVQ4 and NVQ5). Household income was measured using the Organisation for Economic Cooperation and Development income-weighted quintiles (Q1—Lowest, Q2, Q3, Q4 and Q5—Highest); for household tenure ‘own’ (outright, with mortgage/loan and shared equity), ‘social rent’, ‘private rent’ and ‘other’ (eg, living with parents and squatting) options were considered. Finally, we added income deprivation from the 2004 English Index of Multiple Deprivation (Q1—Most, Q2, Q3, Q4 and Q5—Least).

### Statistical analysis

To adjust for oversampling and restore sample representativeness following attrition, we applied complex survey weights in all presented analyses, including descriptive statistics. Linear regression models were fitted to estimate the association using the *survey* package^24^ in R V.4.3.0.^25^

Unadjusted and adjusted (for all confounders) coefficients (*b*) with 95% CIs were provided, and estimates were expressed as risk difference (absence) and SD change (attainment). Effect modifications by sex and household income quintiles were tested by adding an interaction term to the adjusted models; interactions’ overall p values were determined using the Wald test. To reduce Type I error due to a large number of interaction tests for authorised and unauthorised absences (ie, annual data), we provided false discovery rate (FDR) adjusted p values.^26^

We ran four sensitivity analyses. First, instead of defining SGA by a 10% threshold, we reran models with a categorical variable (ie, average-for-gestational-age (>10%), small-for-gestational-age (3%–10%) and very-small-for-gestational-age (<3%)) (*S1*). Second, although the proportion of covariates missing was very low (<3%), we imputed them using multiple imputations by chained equations; estimates across 10 imputed datasets were pooled using Rubin’s rule (*S2*). Third, as newborns delivered preterm are usually smaller, we restricted the sample to term-only births (≥37 weeks) (*S3*). Last, we provided findings for persistent school absences (missing ≥10% of possible sessions), an important classification for educational policy^22^ (*S4*).

## Results

Out of 13 197 eligible individuals participating in Sweep 4, 8290 were living in England. 94% of them had consented to educational data linkage. Two analytical samples were created: one for school absence (n=6659) and one for educational attainment (n=6204) (online supplemental figure 2). Characteristics for the analytical samples are in table 1; to support comparison with the age-specific target population, we provided descriptives for all English MCS participants from Sweep 4. These suggested almost identical birth outcomes across the samples, with some minor differences related to sex, household tenure, household income and area-level income deprivation (table 1). Pupils born SGA versus average-for-gestational-age differed across almost all covariates (online supplemental table 1).

**Table 1:** Weighted sample characteristics

	English sample*(n=8290)	Absence sample(n=6659)	Attainment sample(n=6204)
Sex, % (n)			
Female	48.8 (4105)	49.0 (3293)	50.2 (3139)
Male	51.2 (4184)	51.0 (3366)	49.8 (3065)
Ethnic group, % (n)			
Black or Black British	3.8 (396)	3.2 (264)	3.2 (255)
Indian	2.3 (312)	2.0 (232)	2.1 (224)
Mixed	3.8 (312)	3.5 (233)	3.3 (204)
Pakistani and Bangladeshi	5.6 (795)	4.9 (585)	5.1 (553)
White	83.1 (6304)	84.9 (5233)	85.0 (4862)
Other ethnic groups	1.5 (146)	1.4 (112)	1.4 (106)
Partnership status, % (n)			
Single parent	14.9 (1117)	15.0 (897)	13.5 (762)
Living with partner	85.1 (7173)	85.0 (5762)	86.5 (5442)
Maternal age at birth (years), mean±SD	28.5±5.9	28.3±5.9	28.4±5.8
Maternal smoking, % (n)			
Never smoked	51.5 (4646)	49.9 (3636)	51.3 (3474)
Smoked before pregnancy but reduced/quit	27.5 (2024)	29.3 (1738)	28.2 (1557)
Smoked during pregnancy	6.2 (450)	6.5 (381)	5.9 (320)
Smoked in other times	14.8 (1151)	14.3 (904)	14.6 (853)
Number of siblings			
None	42.1 (3436)	41.9 (2573)	41.2 (2709)
One	36.2 (2924)	36.8 (2216)	36.8 (2381)
Two or more	21.7 (1930)	21.3 (1415)	21.1 (1569)
Highest household education, % (n)			
No education	9.6 (806)	9.4 (617)	8.1 (511)
Overseas only	2.0 (203)	2.1 (169)	1.9 (152)
NVQ level 1	6.2 (487)	6.6 (412)	6.1 (357)
NVQ level 2	25.5 (2036)	27.6 (1780)	27.4 (1645)
NVQ level 3	15.6 (1267)	16.4 (1069)	16.6 (1003)
NVQ level 4	34.8 (2919)	33.1 (2256)	34.7 (2185)
NVQ level 5	6.3 (571)	4.8 (356)	5.1 (351)
Household income, % (n)			
Q1—Lowest income quintile	20.8 (1750)	21.1 (1403)	18.9 (1191)
Q2	20.4 (1783)	21.4 (1494)	21.0 (1370)
Q3	20.0 (1595)	21.3 (1375)	22.0 (1315)
Q4	19.1 (1567)	19.9 (1318)	20.9 (1280)
Q5—Highest income quintile	19.5 (1585)	16.2 (1069)	17.3 (1048)
Household tenure, % (n)			
Own	59.7 (5071)	59.1 (4075)	61.6 (3926)
Social rent	25.4 (2011)	26.2 (1635)	24.2 (1421)
Private rent	9.0 (706)	8.9 (559)	8.4 (495)
Other	5.9 (499)	5.9 (390)	5.8 (362)
Area-level income deprivation, % (n)			
Q1—Most deprived	26.0 (2750)	26.2 (2204)	24.8 (1983)
Q2	21.0 (1781)	22.4 (1524)	22.0 (1400)
Q3	19.3 (1436)	19.4 (1170)	20.0 (1128)
Q4	17.2 (1156)	16.5 (895)	16.8 (846)
Q5—Least deprived	16.5 (1142)	15.5 (866)	16.3 (847)
Birth weight in kg, mean±SD	3.4 (0.6)	3.4 (0.6)	3.4 (0.6)
Small-for-gestational-age†			
Average-for-gestational-age (>10%)	93.5 (7499)	93.3 (6161)	93.5 (5754)
Small-for-gestational-age (≤10%)	6.5 (595)	6.7 (498)	6.5 (450)

Note: Variables were derived from Sweep 1 (age 9 months). Month of birth is not included in the table. Means and percentages are weighted using complex sample weights; frequencies are unweighted. Percentages might not add up to 100 due to rounding.

* Frequencies might not add up to total due to missing values.

† Based on INTERGROWTH-21st standards.^20^

### School attendance

**Figure 1:**
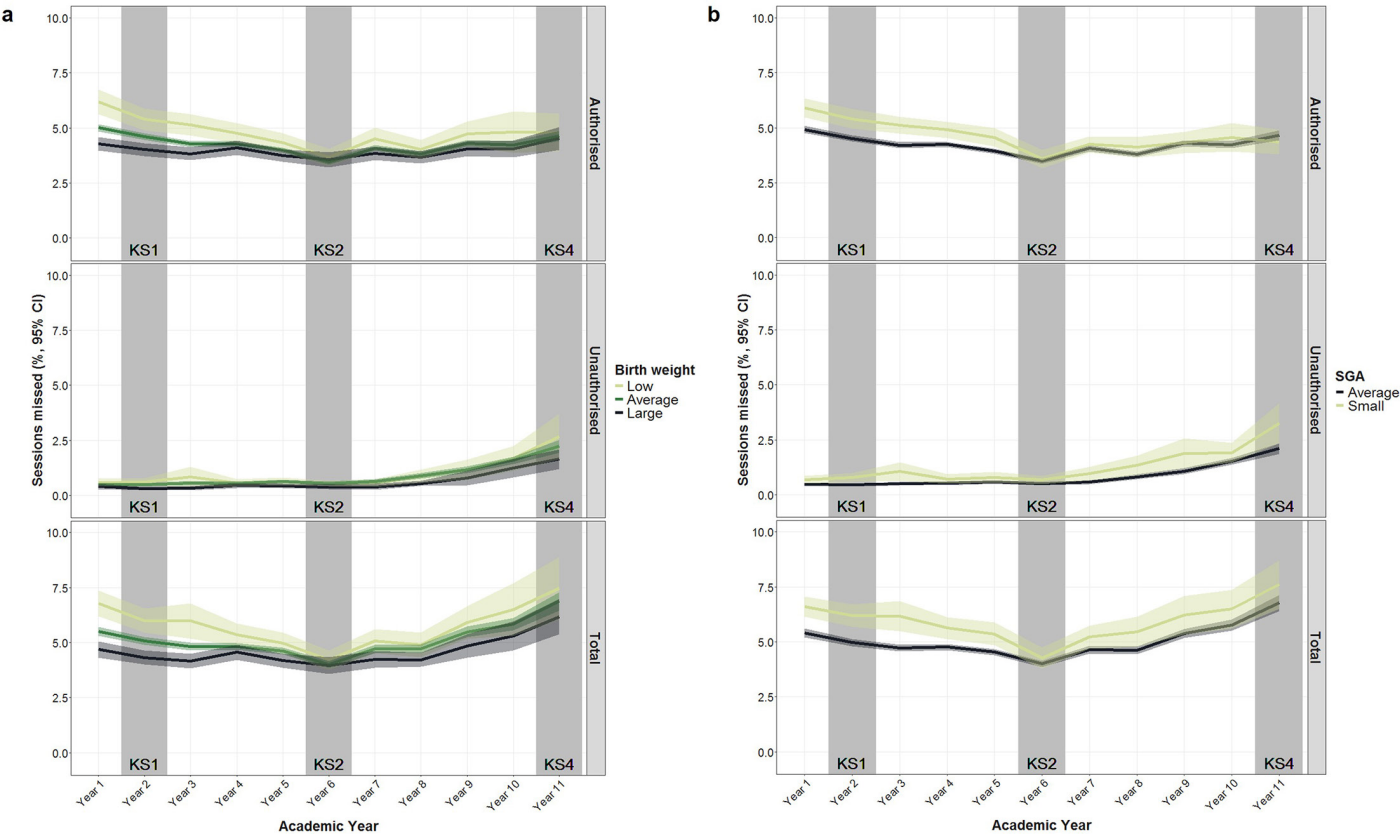
Percentage of missed sessions in Years 1–11 by (a) birth weight and (**b**) SGA. Key stage examinations took place in grey-shaded academic years; birth weight was categorised for this plot into low (<2.5 kg), average (2.5–3.9 kg) and large groups (≥4 kg), n=6659. SGA, small-for-gestational-age.

**Figure 2:**
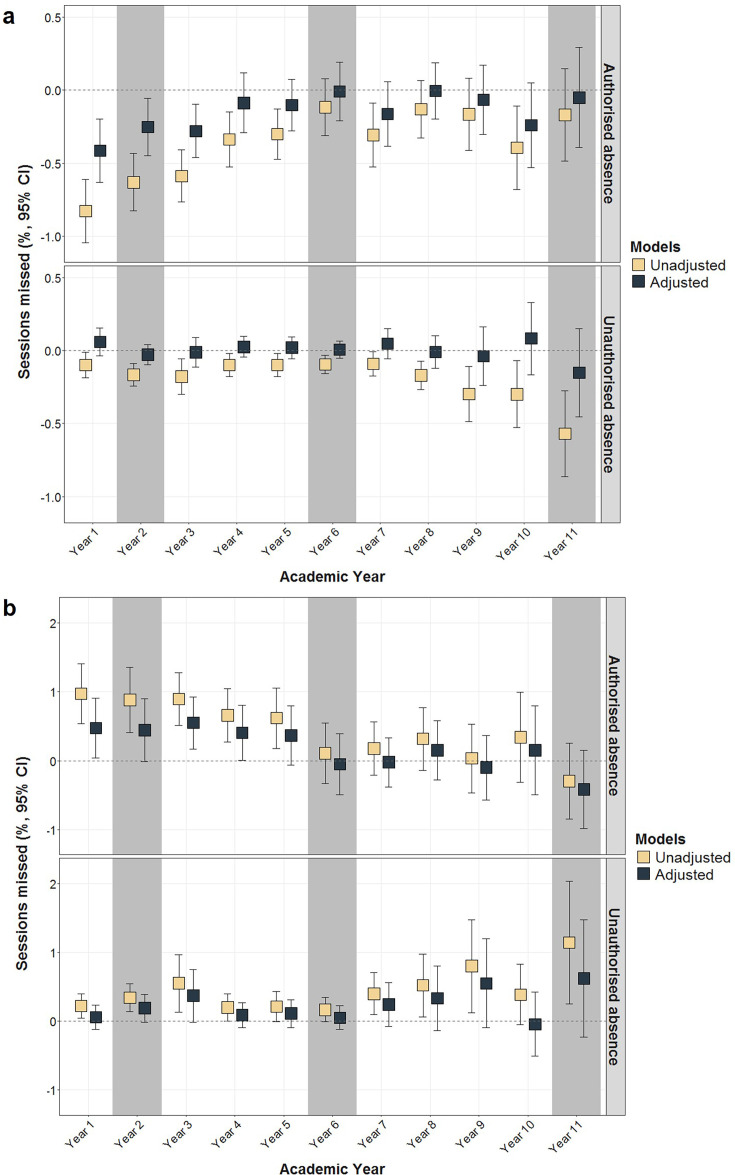
Associations between (a) birth weight, (b) small-for-gestational-age (compared with average-for-gestational-age) and percentage of missed sessions in Years 1–11. Key stage examinations took place in grey-shaded academic years. Linear regressions with complex survey weights were fitted separately for authorised and unauthorised absences; in addition to unadjusted associations, plots present estimates controlled for sex, month of birth, ethnic groups, partnership status, maternal age at birth, maternal smoking, number of siblings, household tenure, highest household education attainment, household income and area-level income deprivation, n=6659.

As shown in figure 1, children born with lower BW or SGA had more authorised absences during primary school (especially Years 1–4); although the percentage of sessions missed during secondary school was also somewhat elevated, CIs were usually overlapping. Moreover, figure 1a suggested a linear relationship between BW and absence. The mean numbers of missed sessions due to authorised and unauthorised absences are in online supplemental table 2; SGA pupils missed an average of 197.9 sessions (5.93% of total; 95% CI 5.53 to 6.33), while average-for-gestational-age children missed 169.8 sessions (5.05% of total; 95% CI 4.91 to 5.20) during 11 years of compulsory education (note: absences are counted during the first five half terms). In the adjusted models, each 1 kg increase in BW was associated with a −0.41% (95% CI −0.63 to –0.20), −0.25% (95% CI −0.45 to –0.06) and −0.28% (95% CI −0.46 to –0.10) reduction in authorised absences in Year 1, Year 2 and Year 3, respectively (figure 2; online supplemental table 3). Being born SGA (vs average-for-gestational-age) increased the percentage of authorised absences by 0.47% (95% CI 0.04 to 0.91), 0.55% (95% CI 0.17 to 0.93) and 0.40% (95% C: 0.00 to 0.80) in Year 1, Year 3 and Year 4, respectively. Neither BW nor SGA was associated with unauthorised absences in the adjusted models (figure 2; Online supplemental table 3).

### Educational attainment

Children born with lower BW or SGA had lower English and Maths test scores (online supplemental table 4), which remained significant even after adjusting for all confounders (table 2): 1 kg increase in BW was associated with 0.10, 0.14 and 0.10 SD higher English and 0.15, 0.16 and 0.11 SD higher Maths scores in KS1, KS2 and KS4 tests, respectively. Being born SGA (vs average-for-gestational-age) was associated with −0.21 to –0.22 and −0.16 SD lower English and −0.26 to –0.21 and −0.20 SD lower Maths scores in KS1, KS2 and KS4 tests, respectively. Associations were also significant for KS4 A8 scores (BW: 0.09 SD and SGA: −0.17 SD). In a post hoc analysis, we explored whether associations remained significant for KS2 and KS4 English and Maths test scores after adjusting for prior scores from the same subject. This suggested significant attenuation with only BW remaining associated with KS2 test scores (English: 0.07 SD and Maths: 0.05 SD; table 2).

**Table 2:** Associations between birth weight, size-for-gestational age and standardised test scores at each key stage

	Unadjusted			Adjusted			Adjusted for prior attainment		
	b	95% CI	P value	b	95% CI	P value	b	95% CI	P value
Birth weight (per 1 kg)									
Key Stage 1 (age 7)									
English (Reading)	0.14	0.09 to 0.19	<0.001	0.10	0.06 to 0.14	<0.001	NA		
Maths	0.22	0.17 to 0.26	<0.001	0.15	0.11 to 0.18	<0.001	NA		
Key Stage 2 (age 11)									
English	0.17	0.12 to 0.22	<0.001	0.14	0.10 to 0.18	<0.001	0.07	0.04 to 0.10	<0.001
Maths	0.23	0.19 to 0.27	<0.001	0.16	0.12 to 0.20	<0.001	0.05	0.02 to 0.08	<0.001
Key Stage 4 (age 16)									
English	0.12	0.08 to 0.17	0.003	0.10	0.06 to 0.14	<0.001	0.02	−0.01 to 0.05	0.166
Maths	0.18	0.14 to 0.23	<0.001	0.11	0.08 to 0.15	<0.001	0.00	−0.02 to 0.03	0.827
Attainment 8	0.14	0.09 to 0.18	<0.001	0.09	0.06 to 0.13	<0.001	NA		
Small-for-gestational-age (vs average-for-gestational-age)									
Key Stage 1 (age 7)									
English (Reading)	−0.29	−0.39 to −0.20	<0.001	−0.21	−0.29 to −0.12	<0.001	NA		
Maths	−0.37	−0.44 to −0.29	<0.001	−0.26	−0.33 to −0.19	<0.001	NA		
Key Stage 2 (age 11)									
English	−0.31	−0.41 to −0.20	<0.001	−0.22	−0.33 to −0.12	<0.001	−0.08	−0.15 to 0.00	0.052
Maths	−0.29	−0.38 to −0.20	<0.001	−0.21	−0.30 to −0.13	<0.001	−0.01	−0.08 to 0.05	0.661
Key Stage 4 (age 16)									
English	−0.24	−0.34 to −0.14	<0.001	−0.16	−0.25 to −0.07	0.002	−0.03	−0.10 to 0.04	0.399
Maths	−0.29	−0.38 to −0.20	<0.001	−0.20	−0.28 to −0.12	<0.001	−0.06	−0.12 to 0.01	0.073
Attainment 8	−0.25	−0.33, −0.16	<0.001	−0.17	−0.25, −0.09	<0.001	NA		

Linear regressions with complex survey weights were fitted, adjusted models controlled for sex, month of birth, ethnic groups, partnership status, maternal age at birth, maternal smoking, number of siblings, household tenure, highest household education attainment, household income and area-level income deprivation. The last model adjusted also for standardised test scores from the prior Key Stage (ie, Key Stage 2 scores are adjusted for Key Stage 1 scores, Key Stage 4 scores are adjusted for Key Stage 2 scores). n=6204.

NA, not applicable.

### Effect modification by sex and household income

**Figure 3:**
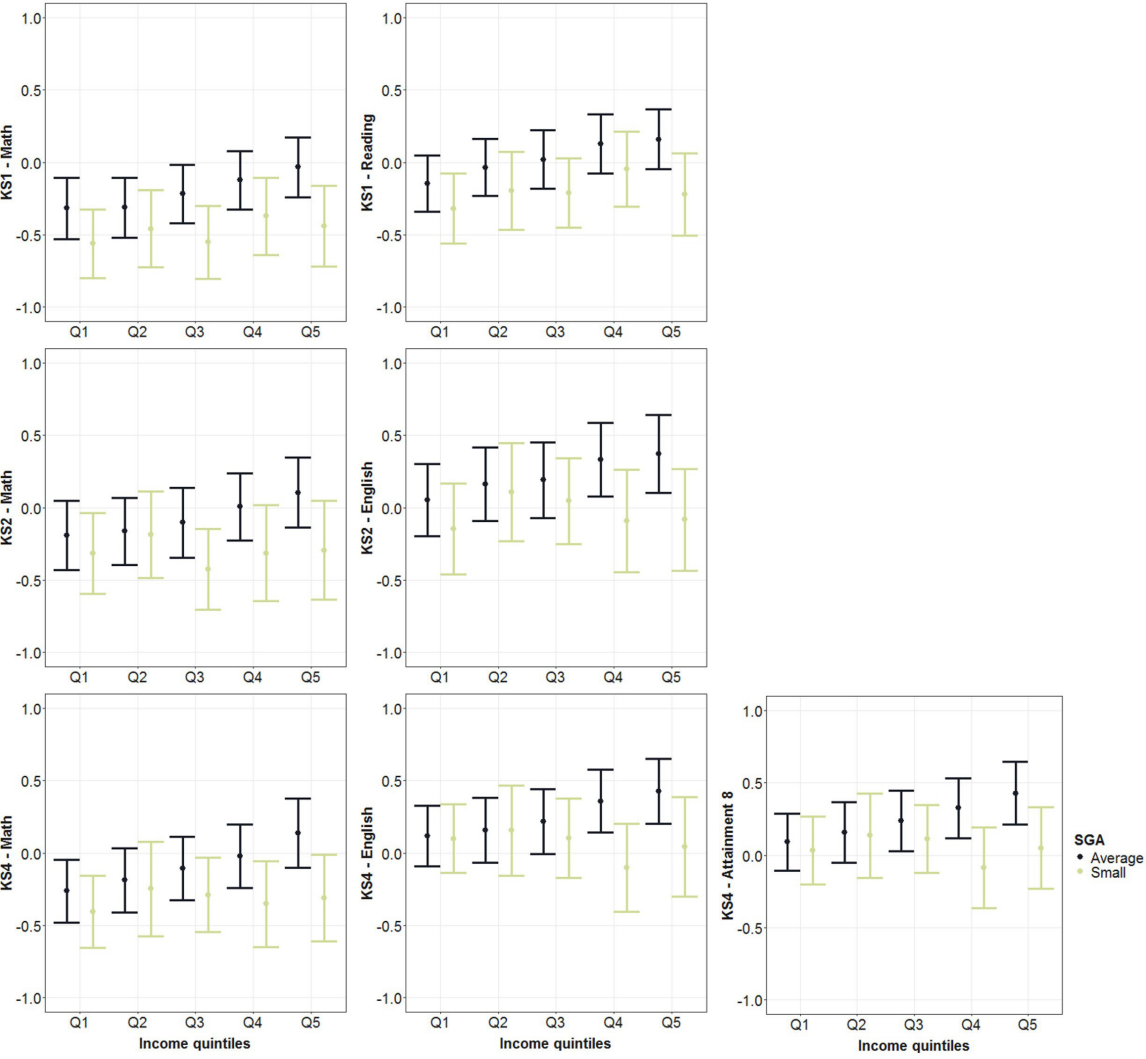
Predicted values of standardised test scores for pupils with average and small-for-gestational-age birth weights across different household income quintiles (Q1–Lowest; Q2; Q3; Q4 and Q5–Highest). Linear regressions with complex survey weights were fitted, and models controlled for sex, month of birth, ethnic groups, partnership status, maternal age at birth, maternal smoking, number of siblings, household tenure, highest household education attainment, and area-level income deprivation. n=6204. KS, Key Stage; SGA, small-for-gestational-age.

Infants in the lowest income households were double as likely to be SGA than infants in the highest income households (9.0% vs 4.2%) (online supplemental table 5), and they also had a greater number of absences (online supplemental figure 3) and lower test scores (online supplemental table 6). There were some differences in attainment between boys and girls, with girls outperforming boys except for Maths (online supplemental tables 5 and 7 and figure 4). Significant effect modification was found between size-for-gestation, household income and educational attainment (online supplemental tables 8–10): for KS2 English and Maths, KS4 English and A8 test results. To better understand effect modification (online supplemental figure 5), we plotted predicted standardised test scores by size-for-gestation and household income (figure 3): for children born average-for-gestational-age, the test performance increased stepwise by each income quintile; however, income-related health inequalities were less apparent for children born SGA.

### Sensitivity analyses

S1: associations with absence and attainment were strongest among children born very-SGA (<3%) (online supplemental figures 6 and 7). S2: imputing missing data led to the exact same findings (online supplemental tables 11 and 12). S3: after excluding participants born preterm, authorised school absence was still linked to SGA (Year 3) and lower BW (Year 1–3) (online supplemental table 13); association with attainment only marginally changed and remained significant (online supplemental table 14). S4: children born SGA had 1.41 (95% CI 1.09 to 1.83) and 1.34 (95% CI 1.04 to 1.74) higher odds of persistent absence in Year 2 and Year 3, respectively, compared with children born average-for-gestational-age (online supplemental figure 8).

## Discussion

This longitudinal data-linkage study following a cohort across the whole national curriculum in England shows how low BW and size-for-gestation are associated with higher authorised school absences during primary school and lower English and Math test scores. A range of sensitivity analyses confirmed these findings. Furthermore, we also found some evidence for household income modifying the associations between size-for-gestation and school attainment, whereby social inequalities in test scores were not observed among children born SGA.

Medical conditions (illness and medical appointments) constituted the overwhelming majority of absences in England in 2016/2017 (ie, year of GCSE in our cohort), accounting for 84% of authorised absences, 85% in primary and 83% in secondary schools.^27^ As suggested by the *developmental origins of health and diseases* hypothesis, fetal development can have life-long consequences for disease development.^28^ Studies have found that BW is linked to neurodevelopmental outcomes (eg, ADHD^29^), mental health (eg, depression^30^), insulin resistance and type 2 diabetes in childhood^31^ and reduced physical fitness.^32^ Our study showed that associations with authorised absences were only present in primary school and especially during the first 3–4 years, where, in addition to sociodemographic factors, SGA was associated with approximately 1 more day of missed school during each of these academic years. Identifying specific health conditions (eg, by linking health records) responsible for authorised absences among low BW and SGA pupils could help to design interventions to support this vulnerable population. Joined-up support from healthcare and education and understanding how these systems can best work together to support affected children, requires further consideration.

In line with previous studies,^10 11^ we showed that children born with lower BW or SGA had lower English and Maths test scores at age 7, 11 and 16 compared with those born average-for-gestational-age. Adjusted coefficients for SGA were between −0.26 and −0.16 suggesting a small-to-moderate effect size, comparable to the difference in average A8 scores (ie, performance across eight GCSE-level qualifications) between boys and girls. Attenuated associations after adjusting KS2 and KS4 test results with prior scores suggested that disparities across BW and SGA groups were present at the beginning of primary school (KS1) and persisted across compulsory education (KS2 and KS4). In addition, between KS1 and KS2, lower BW was further associated with lower KS2 test scores. Literature suggests that low BW (especially very low: <1500 g) is related to lower cognitive abilities over the life course^33 34^, which is moderate-strongly correlated with educational achievement.^35^ Health problems and chronic conditions,^36^ including mental health difficulties^37^ and uncontrolled asthma,^38^ are associated with school performance, suggesting another plausible pathway between birth outcomes and school attainment. Future studies using linked surveys and administrative data should test specific mediating pathways (eg, cognitive function, physical and mental health) between birth outcomes and attainment, including the role of school absences.^39^

Earlier findings from an Australian data-linkage study suggested stronger effects of BW on academic achievements among females,^10^ which we were unable to confirm. Conflicting findings could be explained by differences in power, type of data, confounder adjustment or education systems. The study also found effect modification between BW and attainment by socioeconomic status,^10^ while evidence from a Swedish register study did not confirm it focusing on preterm birth and attainment.^9^ Our data from England showed effect modification across multiple test scores; however, the predicted relationship was different from our hypothesis. Attainment disadvantage related to size-for-gestational was largest in the highest income groups, which is not unknown.^40^ Predicting the mean test scores by groups showed that performance was comparable across all SGA children, and only average-for-gestational-age children benefitted from higher household income. The percentage of SGA infants was more than double in the lowest, in comparison to the highest income group, and it is plausible that SGA newborns had different underlying conditions across income groups. To confirm these findings and to explore specific diseases related to BW inequalities requires much larger cohorts, likely from linked population administrative data (eg, ECHILD).

### Strengths and limitations

This study benefited from a large and nationally representative sample, a comprehensive follow-up across 11 years, a very high linkage rate and a wide range of key confounders, demonstrating the strengths of linking administrative data to traditional cohorts. Although findings are generalisable for the age-specific English population educated in state-funded schools, there are limitations to consider. First, 6% of the eligible sample did not consent to educational data linkage, and there were minor differences between the eligible and effective samples. Second, despite near-population coverage, the National Pupil Database does not include children from independent/private schools (6% of the total in England),^21^ who have likely different social backgrounds. Third, there was a significant proportion of participants with missing information on school attendance/attainment, which points out that despite the overall high linkage rate, linkage success differed considerably across the specific datasets. Fourth, despite various covariates considered in the models, unmeasured confounding cannot be ruled out (eg, congenital anomalies). Fifth, due to availability, we used absence data for the first five half terms, which might somewhat underestimate absence due to lower attendance during the second half of the summer term (eg, average absence between 2012/2013 and 2016/2017 is 5.5% using five vs 5.7% using six half terms). Lastly, we excluded children who did not sit examinations, or whom the school did not deem as being ready; the percentage of SGA children was somewhat higher in this group than in the analytical sample (8% vs 6.5%).

### Conclusions

Our findings based on a representative English sample suggest that children born with lower BW and SGA had higher authorised absences during primary school. They also had lower English and Maths test scores at ages 7, 11 and 16, with disparities already present at the beginning of primary school. Higher household income did not compensate for educational disadvantages related to size-for-gestational, as test performance was comparable across all SGA children. Future studies should explore specific conditions related to school absences and mediating pathways between birth outcomes and school attainment; cohort data linked with educational records and large administrative datasets can be particularly advantageous. Furthermore, understanding how health and educational systems could work together to support affected children should be considered.

## Supplementary material

10.1136/archdischild-2025-328611 online supplemental file 1

## Data Availability

Data are available in a public, open access repository.
