# Supplementary material for: Birth weight and school absences and attainment: a longitudinal linked cohort study of compulsory schooling in England
Source: Arch Dis Child. 2025 Apr 25;110(6):e328611. doi: 10.1136/archdischild-2025-328611 (PMC12171418; doi:10.1136/archdischild-2025-328611)

**Online Supplement**

**Supplementary Figure 1**: Directed acyclic graph including exposure, outcomes and confounders.

**Supplementary Figure 2:** Flowchart presenting sample selection. Sample characteristics for the grey shaded boxes are presented in Table 1.

**Supplementary Table 1:** Sample characteristics by size-for-gestation.

**Supplementary Table 2:** Number of sessions missed across year 1 to 11.

**Supplementary Table 3:** Associations between (a) birth weight, (b) small-for-gestational-age and percentage of missed sessions in years 1 to 11.

**Supplementary Table 4**: Weighted mean key stage attainments by small-for-gestational-age categories

**Supplementary Table 5:** Weighted percentage for small-for-gestational-age categories in the sample by sex and income groups. Frequencies are unweighted.

**Supplementary Figure 3:** Percentage of missed sessions in years 1 to 11 by income quintiles.

**Supplementary Table 6**: Weighted mean standardised test scores by income quintiles

**Supplementary Figure 4:** Percentage of missed sessions in years 1 to 11 by sex.

**Supplementary Table 7**: Weighted mean standardised test scores by sex

**Supplementary Table 8**: Interaction between birth weight, income quintiles and percentage of missed sessions in years 1 to 11.

**Supplementary Table 9**: Interaction between birth weight, sex and percentage of missed sessions in years 1 to 11.

**Supplementary Table 10**: Interaction between birth weight, sex/income quintiles and key stage school attainments.

**Supplementary Figure 5**: Stratified models showing the associations for small-for-gestational-age and key stage school attainments by income quintiles.

**Supplementary Figure 6**: Associations between small-for-gestational-age categories (average [>10%], small [3-10%] and very small [<3%]) and percentage of missed sessions in years 1 to 11.

**Supplementary Figure 7**: Associations between small-for-gestational-age categories (average [>10%], small [3-10%] and very small [<3%]) and key stage school attainments.

**Supplementary Table 11**: Adjusted associations between (a) birth weight, (b) small-for-gestational-age and percentage of missed sessions in years 1 to 11, after multiple imputations.

**Supplementary Table 12**: Adjusted associations between (a) birth weight, (b) small-for-gestational-age and key stage school attainments, after multiple imputations.

**Supplementary Table 13**: Adjusted associations between (a) birth weight, (b) small-for-gestational-age and percentage of missed sessions in years 1 to 11 in non-preterm births (≥37 weeks).

**Supplementary Table 14**: Adjusted associations between (a) birth weight, (b) small-for-gestational-age and key stage school attainments in non-preterm births (≥37 weeks).

**Supplementary Figure 8**: Associations between (a) birth weight, (b) small-for-gestational-age (compared to average-for-gestational-age) and persistent school absences (>10% of session missed).

**Supplementary Figure 1**: Directed acyclic graph including exposure, outcomes and confounders. Graph was created using the *daggity.net* website.


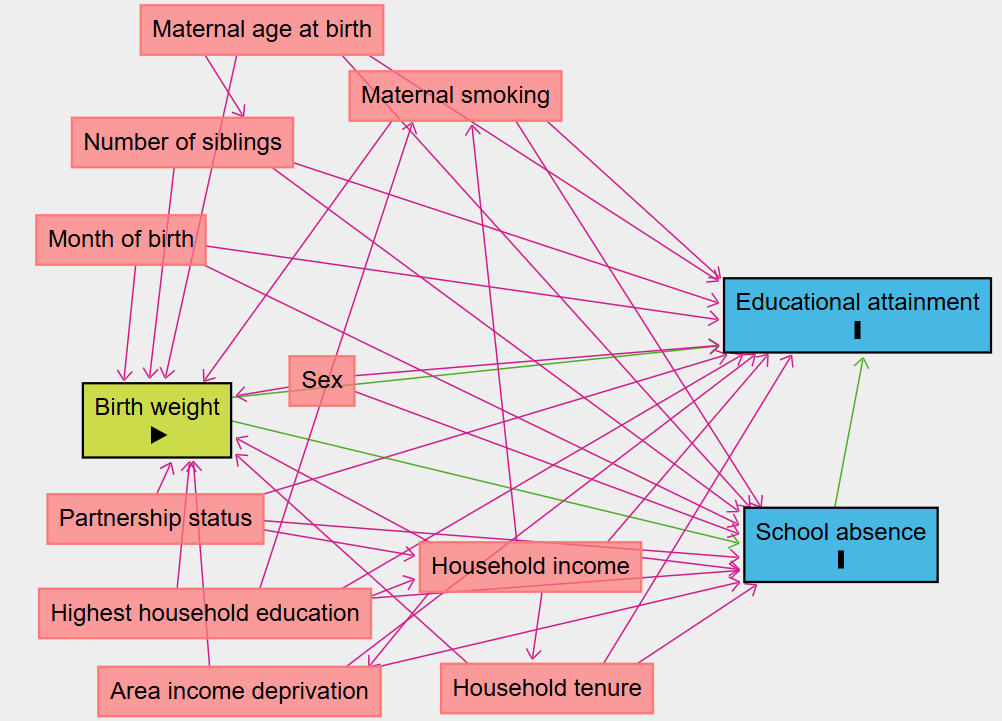


**Supplementary Figure 2:** Flowchart presenting sample selection. Sample characteristics for the grey shaded boxes are presented in Table 1.

**
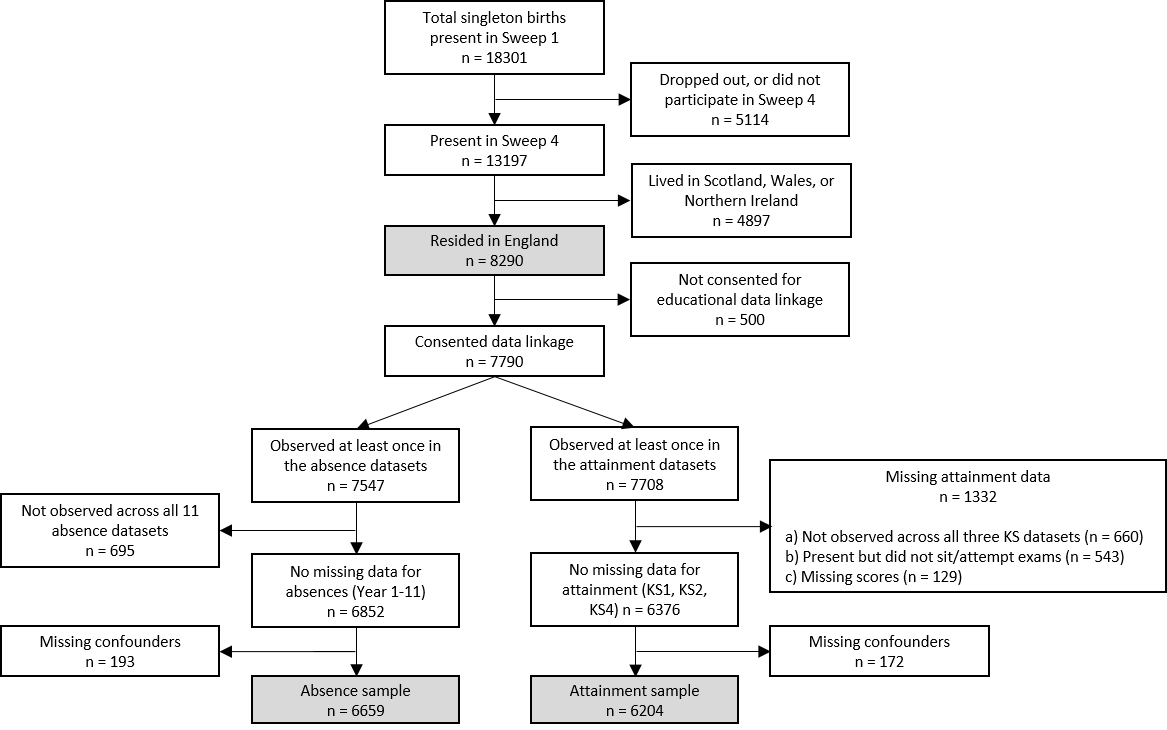
**

**Supplementary Table 1:** Sample characteristics by size-for-gestation.

|  | **Absence sample**  (n=6659) | **Average-for-gestational-age** (n=6161) | **Small-for-gestational-age**  (n=498) |
| --- | --- | --- | --- |
| Sex, % (n) |  |  |  |
| Female | 49.0 (3293) | 48.9 (3038) | 50.7 (255) |
| Male | 51.0 (3366) | 51.1 (3123) | 49.3 (243) |
| Ethnic group, % (n) |  |  |  |
| Black or Black British | 3.2 (264) | 3.1 (239) | 4.7 (25) |
| Indian | 2.0 (232) | 1.8 (187) | 5.3 (45) |
| Mixed | 3.5 (233) | 3.4 (210) | 5.0 (23) |
| Pakistani and Bangladeshi | 4.9 (585) | 4.6 (501) | 10.1 (84) |
| White | 84.9 (5233) | 85.9 (4932) | 71.9 (301) |
| Other Ethnic group | 1.4 (112) | 1.3 (92) | 2.9 (20) |
| Partnership status, % (n) |  |  |  |
| Single Parent | 15.0 (897) | 14.7 (811) | 19.1 (86) |
| Living with partner | 85.0 (5762) | 85.3 (5350) | 80.9 (412) |
| Maternal age at birth (years), mean ± SD | 28.3 ± 5.9 | 28.3 ± 5.9 | 27.7 ± 6.0 |
| Maternal smoking, % (n) |  |  |  |
| Never smoked | 49.9 (3636) | 50.4 (3382) | 41.7 (254) |
| Smoked before pregnancy but reduced/quit | 29.3 (1738) | 28.8 (1589) | 35.6 (149) |
| Smoked during pregnancy | 6.5 (381) | 6.1 (330) | 12.8 (51) |
| Smoked in other times | 14.3 (904) | 14.6 (860) | 10.0 (44) |
| Number of siblings |  |  |  |
| None | 41.9 (2573) | 40.5 (2465) | 49.9 (244) |
| One | 36.8 (2216) | 37.4 (2248) | 28.5 (133) |
| Two or more | 21.3 (1415) | 22.1 (1448) | 21.5 (121) |
| Highest household education, % (n) |  |  |  |
| No education | 9.4 (617) | 9.0 (546) | 15.1 (71) |
| Overseas only | 2.1 (169) | 2.0 (149) | 3.2 (20) |
| NVQ level 1 | 6.6 (412) | 6.5 (369) | 8.2 (43) |
| NVQ level 2 | 27.6 (1780) | 27.4 (1639) | 30.0 (141) |
| NVQ level 3 | 16.4 (1069) | 16.5 (999) | 14.7 (70) |
| NVQ level 4 | 33.1 (2256) | 33.6 (2118) | 26.5 (138) |
| NVQ level 5 | 4.8 (356) | 5.0 (341) | 2.2 (15) |
| Household income, % (n) |  |  |  |
| Q1 – Lowest income quintile | 21.1 (1403) | 20.6 (1252) | 28.3 (151) |
| Q2 | 21.4 (1494) | 21.3 (1373) | 23.0 (121) |
| Q3 | 21.3 (1375) | 21.3 (1276) | 22.0 (99) |
| Q4 | 19.9 (1318) | 20.1 (1242) | 16.7 (76) |
| Q5 – Highest income quintile | 16.2 (1069) | 16.7 (1018) | 10.1 (51) |
| Household tenure, % (n) |  |  |  |
| Own | 59.1 (4075) | 59.6 (3807) | 51.0 (268) |
| Social rent | 26.2 (1635) | 25.9 (1497) | 30.6 (138) |
| Private rent | 8.9 (559) | 8.8 (509) | 10.0 (50) |
| Other | 5.9 (390) | 5.7 (348) | 8.3 (42) |
| Area-level income deprivation, % (n) |  |  |  |
| Q1 – Most deprived | 26.2 (2204) | 25.5 (1975) | 35.9 (229) |
| Q2 | 22.4 (1524) | 22.2 (1406) | 25.5 (118) |
| Q3 | 19.4 (1170) | 19.7 (1102) | 15.2 (68) |
| Q4 | 16.5 (895) | 16.8 (854) | 11.8 (41) |
| Q5 – Least deprived | 15.5 (866) | 15.7 (824) | 11.6 (42) |

*Note*: Variables were derived from Sweep 1 (age 9 months). Month of birth, a confounder for all presented analyses, is not included in the table. Means and %s are weighted using complex sample weights; frequencies are unweighted. Percentages might not add up to 100 due to rounding error.

**Supplementary Table 2:** Number of sessions missed across year 1 to 11.

| **Year** | **Total number of sessions missed** | | **Percentage of sessions missed** | |
| --- | --- | --- | --- | --- |
|  | Average | 95% CI | Average | 95% CI |
| Year 1 (2006/2007) | 16.89 | 16.30, 17.47 | 5.49 | 5.29, 5.68 |
| Year 2 (2007/2008) | 15.42 | 14.92, 15.92 | 5.05 | 4.89, 5.22 |
| Year 3 (2008/2009) | 14.69 | 14.21, 15.17 | 4.82 | 4.65, 4.99 |
| Year 4 (2009/2010) | 14.83 | 14.40, 15.26 | 4.83 | 4.68, 4.97 |
| Year 5 (2010/2011) | 13.92 | 13.53, 14.32 | 4.59 | 4.45, 4.73 |
| Year 6 (2011/2012) | 12.65 | 12.22, 13.09 | 4.02 | 3.87, 4.16 |
| Year 7 (2012/2013) | 14.14 | 13.62, 14.66 | 4.68 | 4.51, 4.86 |
| Year 8 (2013/2014) | 14.04 | 13.51, 14.56 | 4.67 | 4.49, 4.86 |
| Year 9 (2014/2015) | 16.58 | 15.93, 17.23 | 5.44 | 5.22, 5.66 |
| Year 10 (2015/2016) | 18.04 | 17.28, 18.80 | 5.82 | 5.57, 6.07 |
| Year 11 (2016/2017) | 20.52 | 19.45, 21.59 | 6.83 | 6.47, 7.19 |
| **Total (Year 1-11)** | 171.72 | 166.97, 176.48 | 5.11 | 4.97, 5.26 |

Note: missed sessions were recorded by the Department for Education during the first 5 half term. n = 6659

**Supplementary Table 3:** Associations between (a) birth weight, (b) small-for-gestational-age and percentage of missed sessions in years 1 to 11.

|  | **Unadjusted** | | |  | **Adjusted** | | |
| --- | --- | --- | --- | --- | --- | --- | --- |
|  | b | 95% CI | *p* |  | b | 95% CI | *p* |
| **(A) Birth Weight** (per 1kg) | | | | | | | |
| Authorised absences |  |  |  |  |  |  |  |
| Year 1 | -0.83 | -1.04, -0.61 | <0.001 |  | -0.41 | -0.63, -0.20 | <0.001 |
| Year 2 | -0.63 | -0.83, -0.43 | <0.001 |  | -0.25 | -0.45, -0.06 | 0.012 |
| Year 3 | -0.59 | -0.77, -0.41 | <0.001 |  | -0.28 | -0.46, -0.10 | 0.003 |
| Year 4 | -0.34 | -0.53, -0.15 | 0.001 |  | -0.09 | -0.29, 0.12 | 0.403 |
| Year 5 | -0.30 | -0.47, -0.13 | 0.001 |  | -0.10 | -0.28, 0.08 | 0.258 |
| Year 6 | -0.12 | -0.31, 0.08 | 0.243 |  | -0.01 | -0.21, 0.19 | 0.939 |
| Year 7 | -0.31 | -0.52, -0.09 | 0.006 |  | -0.16 | -0.38, 0.06 | 0.146 |
| Year 8 | -0.13 | -0.32, 0.07 | 0.195 |  | 0.00 | -0.20, 0.19 | 0.974 |
| Year 9 | -0.17 | -0.41, 0.08 | 0.190 |  | -0.06 | -0.30, 0.17 | 0.592 |
| Year 10 | -0.39 | -0.68, -0.11 | 0.007 |  | -0.24 | -0.53, 0.05 | 0.104 |
| Year 11 | -0.17 | -0.48, 0.15 | 0.293 |  | -0.05 | -0.39, 0.29 | 0.775 |
| Unauthorised absences |  |  |  |  |  |  |  |
| Year 1 | -0.10 | -0.19, -0.01 | 0.027 |  | 0.06 | -0.04, 0.15 | 0.217 |
| Year 2 | -0.17 | -0.25, -0.09 | <0.001 |  | -0.03 | -0.10, 0.04 | 0.426 |
| Year 3 | -0.18 | -0.03, -0.06 | 0.004 |  | -0.01 | -0.11, 0.09 | 0.800 |
| Year 4 | -0.10 | -0.18, -0.02 | 0.014 |  | 0.03 | -0.05, 0.10 | 0.489 |
| Year 5 | -0.10 | -0.18, -0.02 | 0.016 |  | 0.02 | -0.06, 0.09 | 0.604 |
| Year 6 | -0.10 | -0.16, -0.03 | 0.003 |  | 0.01 | -0.05, 0.06 | 0.864 |
| Year 7 | -0.09 | -0.17, -0.01 | 0.032 |  | 0.05 | -0.06, 0.15 | 0.368 |
| Year 8 | -0.17 | -0.27, -0.07 | 0.001 |  | -0.01 | -0.12, 0.10 | 0.859 |
| Year 9 | -0.30 | -0.49, -0.11 | 0.002 |  | -0.04 | -0.24, 0.16 | 0.705 |
| Year 10 | -0.30 | -0.53, -0.07 | 0.011 |  | 0.08 | -0.16, 0.33 | 0.510 |
| Year 11 | -0.57 | -0.86, -0.28 | <0.001 |  | -0.15 | -0.46, 0.15 | 0.323 |
| **(B) Small-for-gestational-age** (<10%) | | | | | | | |
| Authorised absences |  |  |  |  |  |  |  |
| Year 1 | 0.97 | 0.54, 1.41 | <0.001 |  | 0.47 | 0.04, 0.91 | 0.034 |
| Year 2 | 0.88 | 0.41, 1.36 | <0.001 |  | 0.45 | -0.01, 0.90 | 0.056 |
| Year 3 | 0.90 | 0.52, 1.28 | <0.001 |  | 0.55 | 0.17, 0.93 | 0.005 |
| Year 4 | 0.66 | 0.27, 1.05 | 0.001 |  | 0.40 | 0.00, 0.80 | 0.048 |
| Year 5 | 0.62 | 0.18, 1.06 | 0.006 |  | 0.37 | -0.07, 0.80 | 0.098 |
| Year 6 | 0.11 | -0.34, 0.55 | 0.636 |  | -0.05 | -0.05, 0.39 | 0.820 |
| Year 7 | 0.18 | -0.22, 0.57 | 0.377 |  | -0.02 | -0.38, 0.33 | 0.896 |
| Year 8 | 0.32 | -0.14, 0.78 | 0.174 |  | 0.15 | -0.28, 0.58 | 0.485 |
| Year 9 | 0.03 | -0.47, 0.53 | 0.898 |  | -0.10 | -0.57, 0.37 | 0.672 |
| Year 10 | 0.34 | -0.31, 0.99 | 0.306 |  | 0.15 | -0.50, 0.80 | 0.645 |
| Year 11 | -0.30 | -0.85, 0.25 | 0.288 |  | -0.42 | -0.99, 0.15 | 0.147 |
| Unauthorised absences |  |  |  |  |  |  |  |
| Year 1 | 0.22 | 0.04, 0.39 | 0.015 |  | 0.06 | -0.12, 0.23 | 0.534 |
| Year 2 | 0.34 | 0.14, 0.54 | 0.001 |  | 0.19 | -0.01, 0.39 | 0.068 |
| Year 3 | 0.55 | 0.13, 0.97 | 0.010 |  | 0.37 | -0.01, 0.75 | 0.058 |
| Year 4 | 0.20 | 0.00, 0.39 | 0.047 |  | 0.09 | -0.09, 0.27 | 0.339 |
| Year 5 | 0.21 | -0.01, 0.43 | 0.061 |  | 0.11 | -0.09, 0.31 | 0.283 |
| Year 6 | 0.16 | -0.01, 0.34 | 0.068 |  | 0.05 | -0.12, 0.22 | 0.582 |
| Year 7 | 0.40 | 0.09, 0.70 | 0.011 |  | 0.24 | -0.07, 0.56 | 0.132 |
| Year 8 | 0.52 | 0.06, 0.98 | 0.026 |  | 0.33 | -0.14, 0.80 | 0.167 |
| Year 9 | 0.80 | 0.12, 1.48 | 0.021 |  | 0.55 | -0.10, 1.20 | 0.095 |
| Year 10 | 0.38 | -0.05, 0.82 | 0.086 |  | -0.05 | -0.51, 0.42 | 0.845 |
| Year 11 | 1.14 | 0.25, 2.04 | 0.012 |  | 0.62 | -0.23, 1.48 | 0.152 |

Linear regressions with complex survey weights were fitted separately for authorized and unauthorized absences; in addition to unadjusted associations, plots presented associations controlled for sex, month of birth, ethnic groups, partnership status, maternal age at birth, maternal smoking, number of siblings, household tenure, highest household education attainment, household income, and area-level income deprivation. n=6659

**Supplementary Table 4**: Weighted mean key stage attainments by small-for-gestational-age categories

|  | **Average (>10%)** | |  | **Small (≤10%)** | |  | **p-value** |
| --- | --- | --- | --- | --- | --- | --- | --- |
|  | Mean | 95% CI |  | Mean | 95% CI |  |  |
| KS1 - English (Reading) | 0.16 | 0.14, 0.18 |  | -0.17 | -0.25, -0.08 |  | <0.001 |
| KS2 - English | 0.08 | 0.05, 0.10 |  | -0.26 | -0.34, -0.17 |  | <0.001 |
| KS4 - English | 0.10 | 0.07, 0.12 |  | -0.11 | -0.20, -0.03 |  | <0.001 |
| KS1 - Math | 0.15 | 0.13, 0.17 |  | -0.23 | -0.31, -0.15 |  | <0.001 |
| KS2 - Math | 0.09 | 0.07, 0.11 |  | -0.21 | -0.30, -0.12 |  | <0.001 |
| KS4 - Math | 0.13 | 0.11, 0.15 |  | -0.14 | -0.22, -0.05 |  | <0.001 |
| KS4 - Attainment 8 | 0.24 | 0.22, 0.26 |  | 0.02 | -0.06, 0.09 |  | <0.001 |

n = 6204

**Supplementary Table 5:** Weighted percentage for small-for-gestational-age categories in the sample by sex and income groups. Frequencies are unweighted.

|  | **Small-for-gestational age categories** | | |  | **p-value** |
| --- | --- | --- | --- | --- | --- |
|  | Average (>10%) |  | Small (≤10%) |  |  |
| by Sex |  |  |  |  |  |
| Male | 93.5 (2850) |  | 6.5 (215) |  |  |
| Female | 93.0 (2904) |  | 7.0 (235) |  | 0.567 |
| by Income quintiles |  |  |  |  |  |
| Q1 – Lowest income quintile | 91.0 (1065) |  | 9.0 (126) |  |  |
| Q2 | 92.8 (1263) |  | 7.2 (107) |  |  |
| Q3 | 93.1 (1220) |  | 6.9 (95) |  |  |
| Q4 | 94.4 (1206) |  | 5.6 (74) |  |  |
| Q5 – Highest income quintile | 95.8 (1000) |  | 4.2 (48) |  | <0.001 |

n = 6204

**Supplementary Figure 3:** Percentage of missed sessions in years 1 to 11 by income quintiles; n = 6659

**
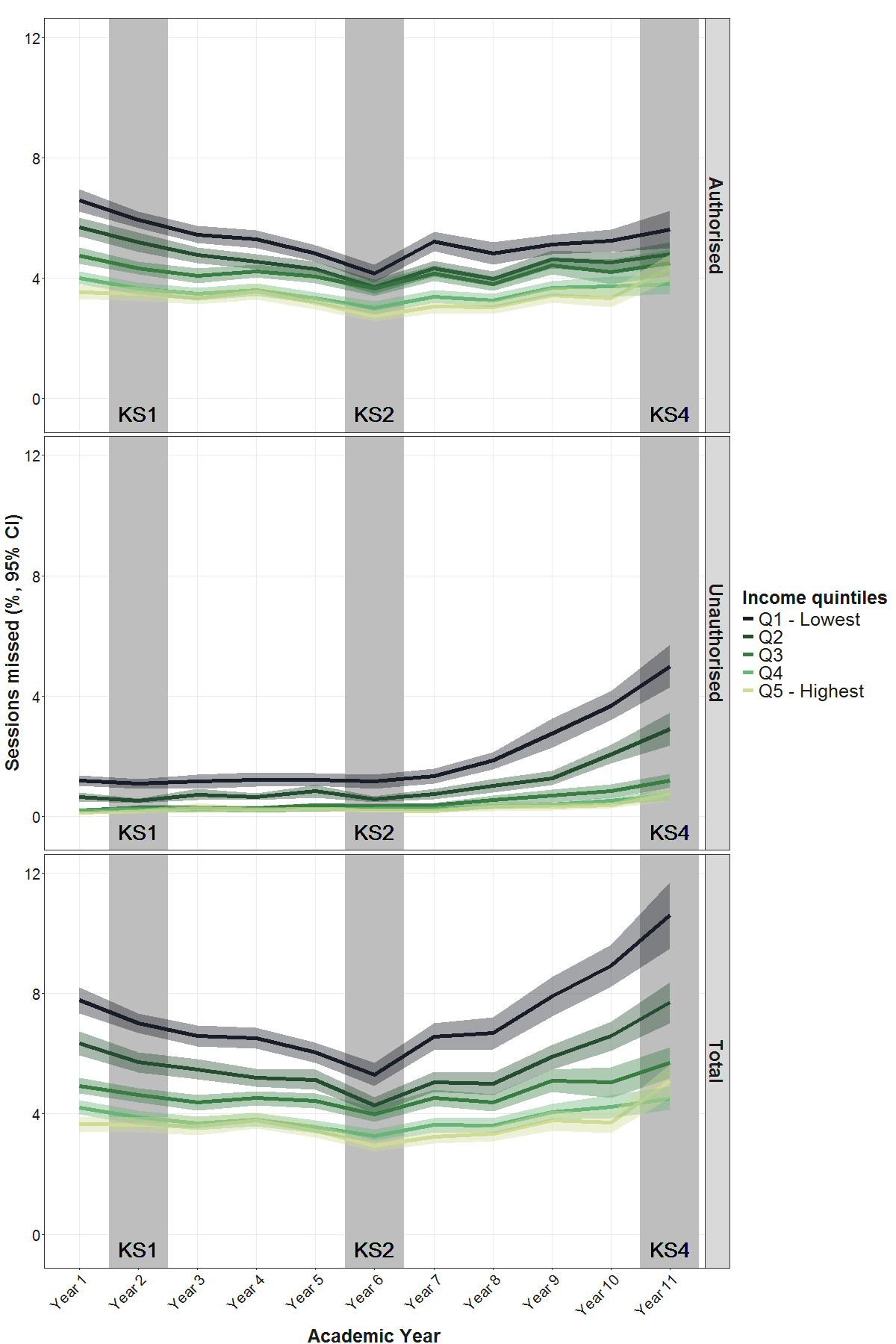
**

**Supplementary Table 6**: Weighted mean standardised test scores by income quintiles

|  | **Q1 - Lowest** | |  | **Q2** | |  | **Q3** | |  | **Q4** | |  | **Q5 - Highest** | |
| --- | --- | --- | --- | --- | --- | --- | --- | --- | --- | --- | --- | --- | --- | --- |
|  | Mean | 95% CI |  | Mean | 95% CI |  | Mean | 95% CI |  | Mean | 95% CI |  | Mean | 95% CI |
| KS1 - English (Reading) | -0.24 | -0.29, -0.18 |  | -0.07 | -0.11, -0.02 |  | 0.14 | 0.09, 0.18 |  | 0.39 | 0.34, 0.43 |  | 0.50 | 0.46, 0.55 |
| KS2 - English | -0.31 | -0.36, -0.26 |  | -0.16 | -0.20, -0.11 |  | 0.04 | -0.02, 0.09 |  | 0.29 | 0.24, 0.34 |  | 0.48 | 0.42, 0.53 |
| KS4 - English | -0.27 | -0.32, -0.22 |  | -0.16 | -0.21, -0.11 |  | 0.04 | -0.01, 0.09 |  | 0.33 | 0.28, 0.39 |  | 0.56 | 0.50, 0.61 |
| KS1 - Math | -0.20 | -0.25, -0.15 |  | -0.09 | -0.13, -0.04 |  | 0.14 | 0.10, 0.19 |  | 0.33 | 0.29, 0.38 |  | 0.48 | 0.44, 0.53 |
| KS2 - Math | -0.24 | -0.29, -0.18 |  | -0.13 | -0.18, -0.08 |  | 0.04 | -0.01, 0.10 |  | 0.27 | 0.22, 0.31 |  | 0.46 | 0.41, 0.51 |
| KS4 - Math | -0.30 | -0.35, -0.25 |  | -0.13 | -0.18, -0.09 |  | 0.10 | 0.05, 0.14 |  | 0.34 | 0.29, 0.39 |  | 0.64 | 0.58, 0.69 |
| KS4 - Attainment 8 | -0.15 | -0.20, -0.11 |  | -0.01 | -0.05, 0.03 |  | 0.21 | 0.17, 0.25 |  | 0.45 | 0.41, 0.50 |  | 0.71 | 0.66, 0.76 |

n = 6204

**Supplementary Figure 4:** Percentage of missed sessions in years 1 to 11 by sex. n = 6659

**
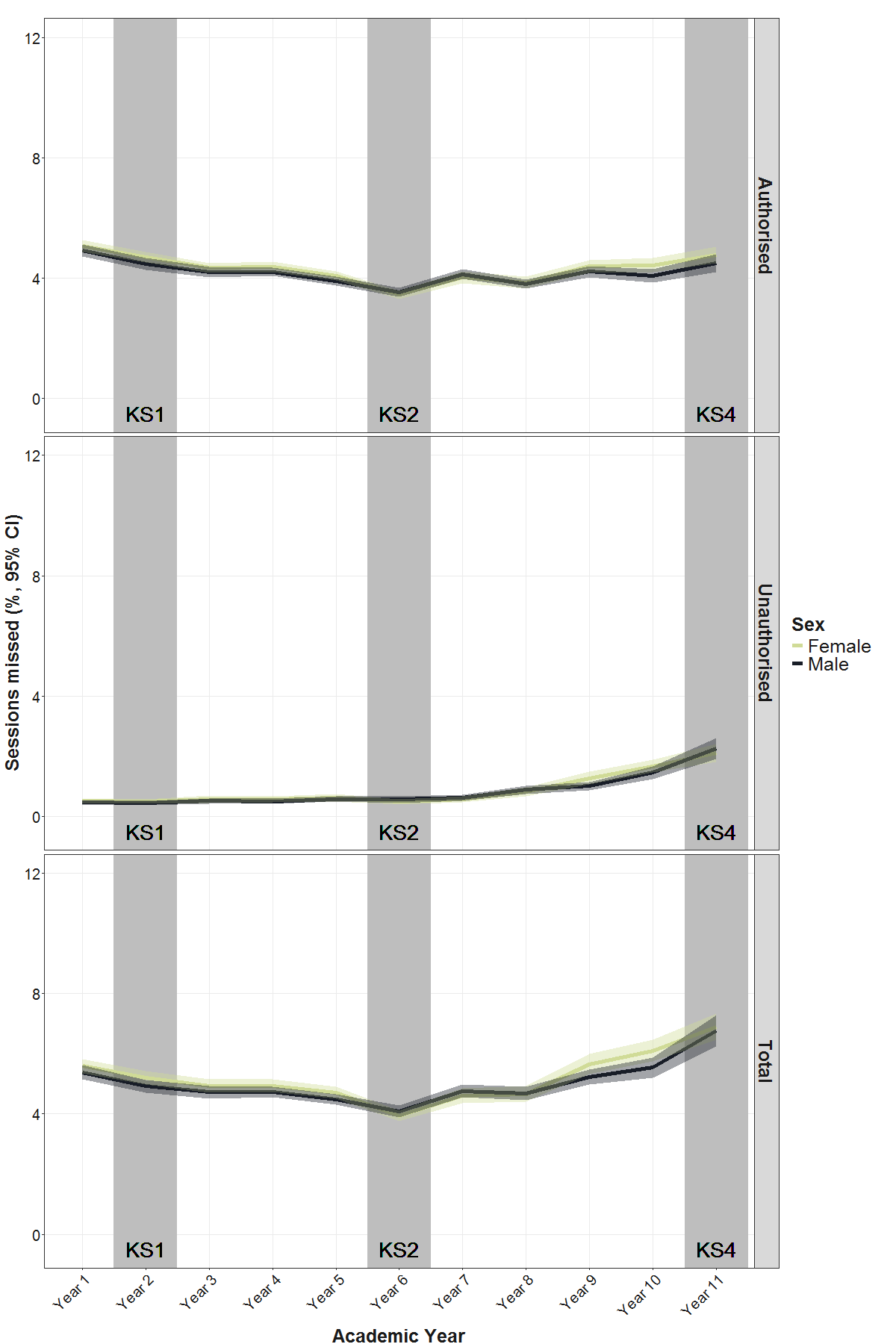
**

**Supplementary Table 7**: Weighted mean standardised test scores by sex

|  | **Male** | |  | **Female** | |  | **p-value** |
| --- | --- | --- | --- | --- | --- | --- | --- |
|  | Mean | 95% CI |  | Mean | 95% CI |  |  |
| KS1 - English (Reading) | 0.03 | 0.00, 0.06 |  | 0.24 | 0.21, 0.27 |  | <0.001 |
| KS2 - English | -0.09 | -0.12, -0.05 |  | 0.19 | 0.16, 0.22 |  | <0.001 |
| KS4 - English | -0.11 | -0.15, -0.08 |  | 0.28 | 0.24, 0.31 |  | <0.001 |
| KS1 - Math | 0.16 | 0.13, 0.20 |  | 0.09 | 0.06, 0.12 |  | 0.006 |
| KS2 - Math | 0.16 | 0.13, 0.19 |  | -0.02 | -0.05, 0.01 |  | <0.001 |
| KS4 - Math | 0.13 | 0.10, 0.17 |  | 0.09 | 0.06, 0.12 |  | 0.321 |
| KS4 - Attainment 8 | 0.13 | 0.10, 0.16 |  | 0.32 | 0.29, 0.35 |  | <0.001 |

n = 6204

**Supplementary Table 8**: Interaction between birth weight, income quintiles and percentage of missed sessions in years 1 to 11.

|  | Authorised | | |  | Unauthorised | | |
| --- | --- | --- | --- | --- | --- | --- | --- |
|  | F-statistics | p-value | p_FDR_ |  | F-statistics | p-value | p_FDR_ |
| Birth weight | | | | | | | |
| Year 1 | 1.313 | 0.267 | 0.857 |  | 1.036 | 0.390 | 0.536 |
| Year 2 | 0.605 | 0.659 | 0.949 |  | 1.190 | 0.316 | 0.497 |
| Year 3 | 3.072 | 0.018 | 0.194 |  | 0.477 | 0.753 | 0.828 |
| Year 4 | 0.304 | 0.875 | 0.949 |  | 0.552 | 0.698 | 0.828 |
| Year 5 | 0.730 | 0.573 | 0.949 |  | 0.302 | 0.877 | 0.877 |
| Year 6 | 1.240 | 0.296 | 0.857 |  | 1.510 | 0.201 | 0.497 |
| Year 7 | 0.370 | 0.830 | 0.949 |  | 1.693 | 0.153 | 0.497 |
| Year 8 | 0.179 | 0.949 | 0.949 |  | 1.915 | 0.110 | 0.497 |
| Year 9 | 0.539 | 0.708 | 0.949 |  | 1.274 | 0.282 | 0.497 |
| Year 10 | 1.036 | 0.390 | 0.857 |  | 1.199 | 0.313 | 0.497 |
| Year 11 | 1.118 | 0.349 | 0.857 |  | 1.606 | 0.175 | 0.497 |
| Small-for-gestational-age (≤10% versus >10%) | | | | | | | |
| Year 1 | 0.977 | 0.422 | 0.795 |  | 0.502 | 0.735 | 0.808 |
| Year 2 | 0.228 | 0.922 | 0.985 |  | 1.583 | 0.180 | 0.782 |
| Year 3 | 1.312 | 0.267 | 0.795 |  | 0.819 | 0.515 | 0.782 |
| Year 4 | 0.721 | 0.578 | 0.795 |  | 0.183 | 0.947 | 0.947 |
| Year 5 | 1.827 | 0.125 | 0.795 |  | 1.006 | 0.406 | 0.782 |
| Year 6 | 1.223 | 0.302 | 0.795 |  | 0.735 | 0.569 | 0.782 |
| Year 7 | 1.171 | 0.325 | 0.795 |  | 1.676 | 0.157 | 0.782 |
| Year 8 | 0.093 | 0.985 | 0.985 |  | 1.335 | 0.258 | 0.782 |
| Year 9 | 0.832 | 0.506 | 0.795 |  | 0.560 | 0.692 | 0.808 |
| Year 10 | 0.871 | 0.482 | 0.795 |  | 0.880 | 0.477 | 0.782 |
| Year 11 | 0.208 | 0.934 | 0.985 |  | 1.141 | 0.338 | 0.782 |

Linear regressions with complex survey weights were fitted and models were controlled for sex, month of birth, ethnic groups, partnership status, maternal age at birth, maternal smoking, number of siblings, household tenure, highest household education attainment, and area-level income deprivation. F-statistics and p-values (plus false discovery rate [FDR] adjusted p-values) from Wald-test are shown for interaction terms. n = 6659

**Supplementary Table 9**: Interaction between birth weight, sex and percentage of missed sessions in years 1 to 11.

|  | **Authorised** | | |  | **Unauthorised** | | |
| --- | --- | --- | --- | --- | --- | --- | --- |
|  | F-statistics | p-value | p_FDR_ |  | F-statistics | p-value | p_FDR_ |
| Birth weight | | | | | | | |
| Year 1 | 0.924 | 0.338 | 0.984 |  | 1.945 | 0.165 | 0.453 |
| Year 2 | 0.299 | 0.585 | 0.984 |  | 5.298 | 0.022 | 0.247 |
| Year 3 | 0.096 | 0.757 | 0.984 |  | 0.047 | 0.829 | 0.829 |
| Year 4 | 0.383 | 0.537 | 0.984 |  | 1.207 | 0.273 | 0.457 |
| Year 5 | 0.052 | 0.820 | 0.984 |  | 1.121 | 0.291 | 0.457 |
| Year 6 | 0.138 | 0.710 | 0.984 |  | 2.840 | 0.094 | 0.453 |
| Year 7 | 0.072 | 0.789 | 0.984 |  | 0.134 | 0.714 | 0.786 |
| Year 8 | 0.000 | 0.984 | 0.984 |  | 1.508 | 0.221 | 0.457 |
| Year 9 | 0.145 | 0.704 | 0.984 |  | 0.151 | 0.698 | 0.786 |
| Year 10 | 0.001 | 0.978 | 0.984 |  | 0.901 | 0.344 | 0.473 |
| Year 11 | 0.012 | 0.912 | 0.984 |  | 2.250 | 0.135 | 0.453 |
| Small-for-gestational-age (≤10% versus >10%) | | | | | | | |
| Year 1 | 0.040 | 0.841 | 0.935 |  | 0.243 | 0.623 | 0.761 |
| Year 2 | 2.361 | 0.126 | 0.462 |  | 0.013 | 0.909 | 0.909 |
| Year 3 | 2.830 | 0.094 | 0.462 |  | 0.384 | 0.536 | 0.737 |
| Year 4 | 1.162 | 0.283 | 0.596 |  | 3.366 | 0.068 | 0.532 |
| Year 5 | 0.009 | 0.926 | 0.935 |  | 0.390 | 0.533 | 0.737 |
| Year 6 | 0.083 | 0.774 | 0.935 |  | 0.548 | 0.460 | 0.737 |
| Year 7 | 0.294 | 0.588 | 0.924 |  | 0.467 | 0.495 | 0.737 |
| Year 8 | 2.520 | 0.114 | 0.462 |  | 1.780 | 0.184 | 0.674 |
| Year 9 | 0.973 | 0.325 | 0.596 |  | 1.105 | 0.295 | 0.737 |
| Year 10 | 0.007 | 0.935 | 0.935 |  | 2.787 | 0.097 | 0.532 |
| Year 11 | 1.251 | 0.265 | 0.596 |  | 0.150 | 0.699 | 0.769 |

Linear regressions with complex survey weights were fitted and models were controlled for month of birth, ethnic groups, partnership status, maternal age at birth, maternal smoking, number of siblings, household tenure, highest household education attainment, household income, and area-level income deprivation. F-statistics and p-values (plus false discovery rate [FDR] adjusted p-values) from Wald-test are shown for interaction terms. n = 6659

**Supplementary Table 10**: Interaction between birth weight, sex/income quintiles and key stage school attainments.

|  | Sex | |  | Income | |
| --- | --- | --- | --- | --- | --- |
|  | F-statistics | p-value |  | F-statistics | p-value |
| Birth weight | | |  | | |
| KS1 - English (Reading) | 0.115 | 0.735 |  | 0.658 | 0.622 |
| KS2 - English | 0.237 | 0.627 |  | 1.274 | 0.282 |
| KS4 - English | 0.009 | 0.927 |  | 1.499 | 0.204 |
| KS1 - Maths | 1.079 | 0.300 |  | 0.865 | 0.486 |
| KS2 - Maths | 0.117 | 0.733 |  | 0.714 | 0.583 |
| KS4 - Maths | 0.557 | 0.456 |  | 1.567 | 0.185 |
| KS4 - Attainment 8 | 0.009 | 0.923 |  | 2.346 | 0.056 |
| Small-for-gestational-age (≤10% versus >10%) | | |  | | |
| KS1 - English (Reading) | 0.038 | 0.846 |  | 0.868 | 0.485 |
| KS2 - English | 1.306 | 0.255 |  | **3.122** | **0.016** |
| KS4 - English | 0.653 | 0.420 |  | **4.421** | **0.002** |
| KS1 - Maths | 0.022 | 0.883 |  | 1.344 | 0.255 |
| KS2 - Maths | 0.139 | 0.710 |  | **2.481** | **0.045** |
| KS4 - Maths | 1.120 | 0.291 |  | 1.939 | 0.106 |
| KS4 - Attainment 8 | 0.970 | 0.326 |  | **4.180** | **0.003** |

Linear regressions with complex survey weights were fitted and models were controlled for (sex) month of birth, ethnic groups, partnership status, maternal age at birth, maternal smoking, number of siblings, household tenure, highest household education attainment, (household income,) and area-level income deprivation. F-statistics and p-values (plus false discovery rate [FDR] adjusted p-values) from Wald-test are shown for interaction terms. n = 6204

**Supplementary Figure 5**: Stratified models showing the associations for small-for-gestational-age and key stage school attainments by income quintiles. Interactions were evaluated using Wald-test, p-values for interactions are shown in brackets. Linear regressions with complex survey weights were fitted and associations were controlled for sex, month of birth, ethnic groups, partnership status, maternal age at birth, maternal smoking, number of siblings, household tenure, highest household education attainment, and area-level income deprivation. n = 6204


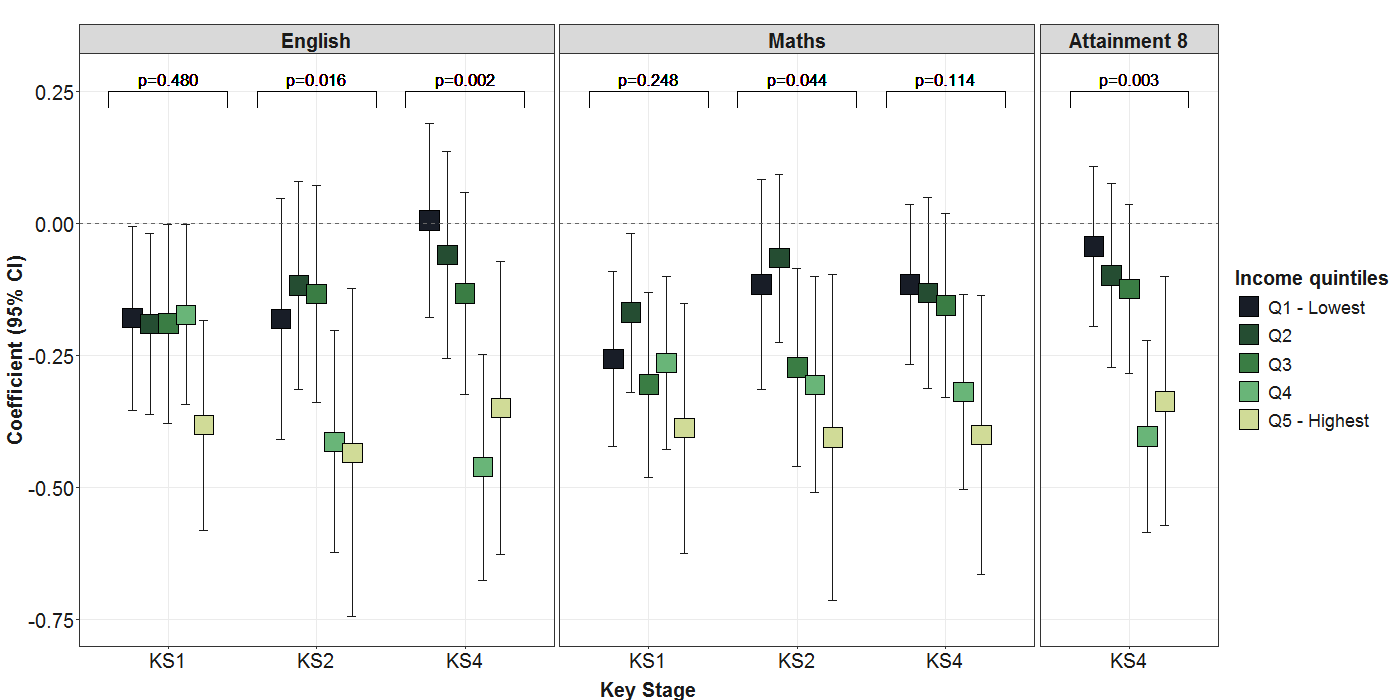


**Supplementary Figure 6**: Associations between small-for-gestational-age categories (average [>10%], small [3-10%] and very small [<3%]) and percentage of missed sessions in years 1 to 11. Linear regressions with complex survey weights are presented separately for authorized and unauthorized absences; associations were controlled for sex, month of birth, ethnic groups, partnership status, maternal age at birth, maternal smoking, number of siblings, household tenure, highest household education attainment, household income, and area-level income deprivation. n = 6659


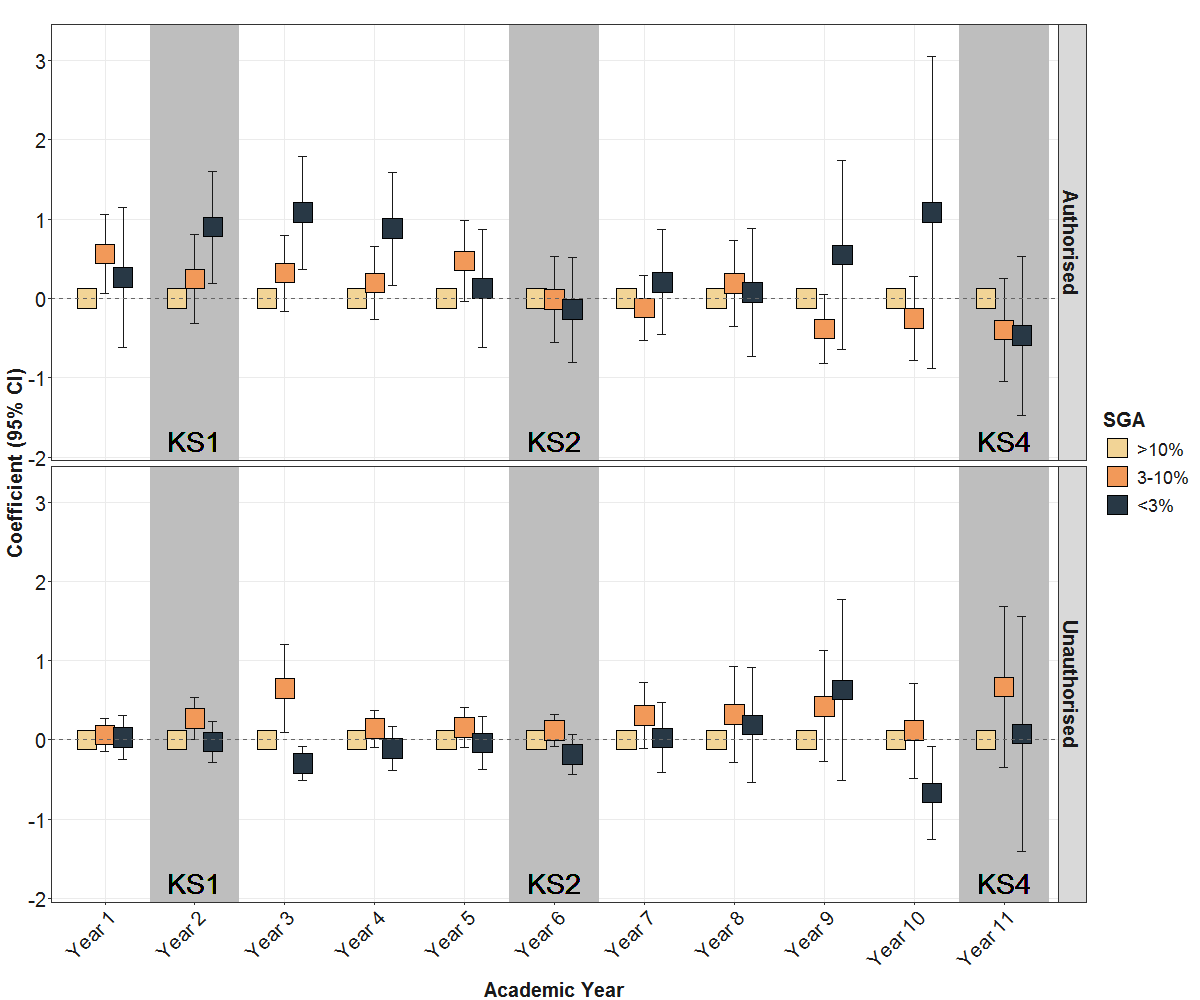


**Supplementary Figure 7**: Associations between small-for-gestational-age categories (average [>10%], small [3-10%] and very small [<3%]) and key stage school attainments. Linear regressions with complex survey weights are presented; associations were controlled for sex, month of birth, ethnic groups, partnership status, maternal age at birth, maternal smoking, number of siblings, household tenure, highest household education attainment, household income, and area-level income deprivation. n = 6204


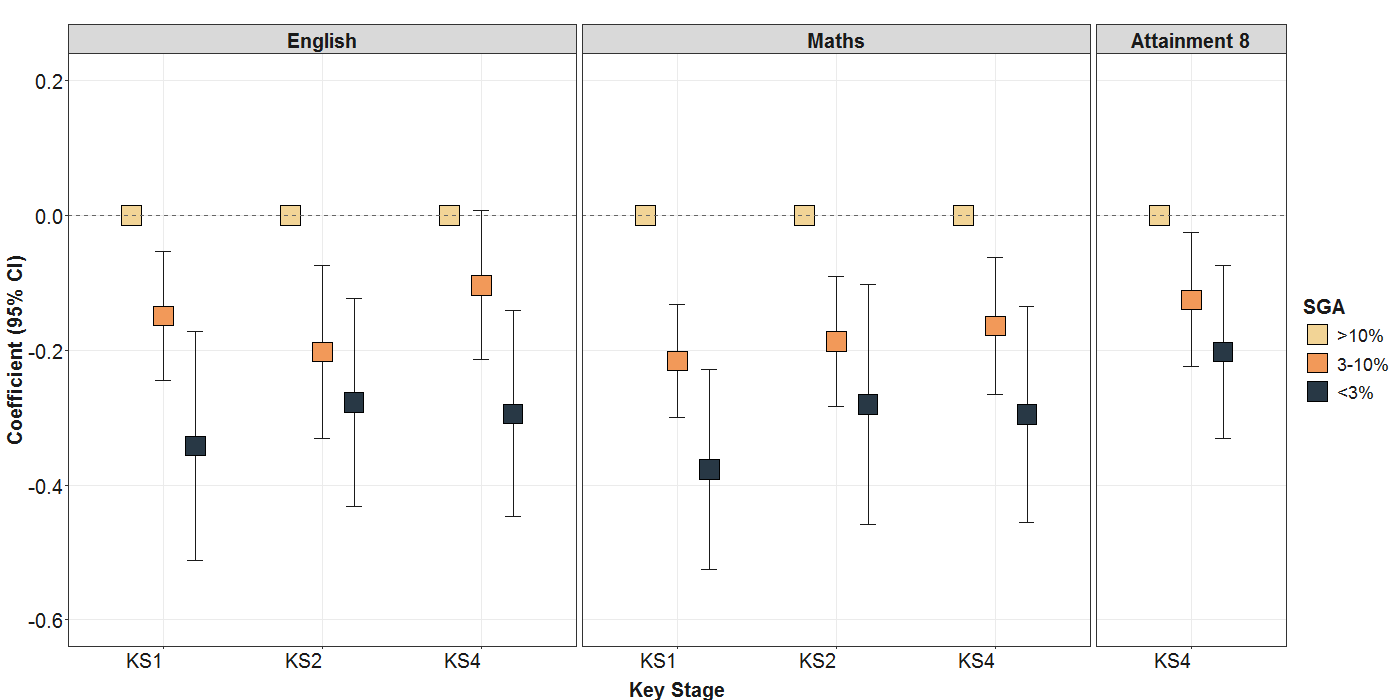


**Supplementary Table 11**: Adjusted associations between (a) birth weight, (b) small-for-gestational-age and percentage of missed sessions in years 1 to 11, after multiple imputations.

|  | **(a) Birth Weight** (per 1kg) | | |  | **(b) Small-for-gestational-age** (≤10% versus >10%) | | |
| --- | --- | --- | --- | --- | --- | --- | --- |
|  | b | 95% CI | *p* |  | b | 95% CI | *p* |
| Authorised absences |  |  |  |  |  |  |  |
| Year 1 | -0.48 | -0.74, -0.23 | <0.001 |  | 0.47 | 0.02, 0.92 | 0.040 |
| Year 2 | -0.25 | -0.45, -0.06 | 0.012 |  | 0.44 | -0.02, 0.91 | 0.058 |
| Year 3 | -0.28 | -0.47, -0.10 | 0.002 |  | 0.55 | 0.16, 0.94 | 0.005 |
| Year 4 | -0.10 | -0.30, 0.11 | 0.365 |  | 0.40 | 0.00, 0.81 | 0.050 |
| Year 5 | -0.11 | -0.29, 0.06 | 0.211 |  | 0.37 | -0.07, 0.80 | 0.100 |
| Year 6 | -0.02 | -0.22, 0.18 | 0.823 |  | -0.05 | -0.50, 0.40 | 0.821 |
| Year 7 | -0.18 | -0.41, 0.04 | 0.102 |  | -0.02 | -0.39, 0.34 | 0.898 |
| Year 8 | 0.01 | -0.19, 0.20 | 0.930 |  | 0.15 | -0.30, 0.60 | 0.505 |
| Year 9 | -0.04 | -0.28, 0.19 | 0.709 |  | -0.10 | -0.58, 0.38 | 0.676 |
| Year 10 | -0.20 | -0.49, 0.09 | 0.182 |  | 0.15 | -0.50, 0.80 | 0.647 |
| Year 11 | 0.02 | -0.34, 0.38 | 0.919 |  | -0.42 | -0.99, 0.15 | 0.150 |
| Unauthorised absences |  |  |  |  |  |  |  |
| Year 1 | 0.06 | -0.04, 0.16 | 0.218 |  | 0.06 | -0.12, 0.23 | 0.538 |
| Year 2 | -0.02 | -0.09, 0.05 | 0.534 |  | 0.19 | -0.02, 0.39 | 0.072 |
| Year 3 | -0.01 | -0.11, 0.09 | 0.847 |  | 0.37 | -0.01, 0.75 | 0.059 |
| Year 4 | 0.03 | -0.05, 0.10 | 0.485 |  | 0.09 | -0.10, 0.28 | 0.355 |
| Year 5 | 0.02 | -0.06, 0.10 | 0.594 |  | 0.11 | -0.10, 0.32 | 0.294 |
| Year 6 | 0.01 | -0.05, 0.07 | 0.674 |  | 0.05 | -0.13, 0.23 | 0.597 |
| Year 7 | 0.07 | -0.04, 0.17 | 0.220 |  | 0.24 | -0.08, 0.56 | 0.136 |
| Year 8 | -0.01 | -0.12, 0.10 | 0.906 |  | 0.33 | -0.14, 0.80 | 0.169 |
| Year 9 | 0.00 | -0.19, 0.20 | 0.964 |  | 0.55 | -0.10, 1.20 | 0.098 |
| Year 10 | 0.10 | -0.15, 0.35 | 0.440 |  | -0.05 | -0.52, 0.42 | 0.847 |
| Year 11 | -0.12 | -0.43, 0.19 | 0.447 |  | 0.62 | -0.25, 1.49 | 0.160 |

Ten datasets were imputed using multiple imputations by changed regressions; linear regressions with complex survey weights adjusted for sex, month of birth, ethnic groups, partnership status, maternal age at birth, maternal smoking, number of siblings, household tenure, highest household education attainment, household income, and area-level income deprivation were fitted and pooled based on Rubin’s rule. n = 6852.

**Supplementary Table 12**: Adjusted associations between (a) birth weight, (b) small-for-gestational-age and key stage school attainments, after multiple imputations.

|  | **(a) Birth Weight** (per 1kg) | | |  | **(b) Small-for-gestational-age** (≤10% versus >10%) | | |
| --- | --- | --- | --- | --- | --- | --- | --- |
|  | b | 95% CI | *p* |  | b | 95% CI | *p* |
| Key Stage 1 |  |  |  |  |  |  |  |
| English (Reading) | 0.10 | 0.06, 0.14 | <0.001 |  | -0.21 | -0.29, -0.12 | <0.001 |
| Maths | 0.15 | 0.11, 0.18 | <0.001 |  | -0.26 | -0.33, -0.19 | <0.001 |
| Key Stage 2 |  |  |  |  |  |  |  |
| English | 0.14 | 0.10, 0.18 | <0.001 |  | -0.22 | -0.33, -0.12 | <0.001 |
| Maths | 0.16 | 0.12, 0.20 | <0.001 |  | -0.22 | -0.30, -0.13 | <0.001 |
| Key Stage 4 |  |  |  |  |  |  |  |
| English | 0.10 | 0.06, 0.14 | <0.001 |  | -0.16 | -0.25, -0.07 | 0.001 |
| Maths | 0.11 | 0.07, 0.15 | <0.001 |  | -0.20 | -0.29, -0.12 | <0.001 |
| Attainment 8 score | 0.09 | 0.06, 0.13 | <0.001 |  | -0.17 | -0.25, -0.09 | <0.001 |

Ten datasets were imputed using multiple imputations by changed regressions; linear regressions with complex survey weights adjusted for sex, month of birth, ethnic groups, partnership status, maternal age at birth, maternal smoking, number of siblings, household tenure, highest household education attainment, household income, and area-level income deprivation were fitted and pooled based on Rubin’s rule. n = 6376.

**Supplementary Table 13**: Adjusted associations between (a) birth weight, (b) small-for-gestational-age and percentage of missed sessions in years 1 to 11 in non-preterm births (≥37 weeks).

|  | **Unadjusted** | | |  | **Adjusted** | | |
| --- | --- | --- | --- | --- | --- | --- | --- |
|  | b | 95% CI | *p* |  | b | 95% CI | *p* |
| **(A) Birth Weight** (per 1kg) | | | | | | | |
| Authorised absences |  |  |  |  |  |  |  |
| Year 1 | -0.78 | -1.03, -0.52 | <0.001 |  | -0.28 | -0.55, -0.02 | 0.036 |
| Year 2 | -0.72 | -0.95, -0.48 | <0.001 |  | -0.24 | -0.47, -0.01 | 0.043 |
| Year 3 | -0.64 | -0.86, -0.42 | <0.001 |  | -0.27 | -0.49, -0.05 | 0.015 |
| Year 4 | -0.41 | -0.64, -0.19 | <0.001 |  | -0.10 | -0.35, 0.14 | 0.394 |
| Year 5 | -0.39 | -0.60, -0.18 | <0.001 |  | -0.14 | -0.35, 0.07 | 0.187 |
| Year 6 | -0.05 | -0.27, 0.17 | 0.665 |  | 0.08 | -0.15, 0.30 | 0.506 |
| Year 7 | -0.23 | -0.46, 0.00 | 0.049 |  | -0.06 | -0.29, 0.17 | 0.634 |
| Year 8 | -0.15 | -0.38, 0.08 | 0.191 |  | 0.00 | -0.21, 0.22 | 0.969 |
| Year 9 | -0.04 | -0.34, 0.26 | 0.782 |  | -0.04 | -0.34, 0.26 | 0.782 |
| Year 10 | -0.51 | -0.88, -0.15 | 0.006 |  | -0.30 | -0.67, 0.06 | 0.104 |
| Year 11 | -0.23 | -0.60, 0.14 | 0.215 |  | -0.09 | -0.49, 0.31 | 0.657 |
| Unauthorised absences |  |  |  |  |  |  |  |
| Year 1 | -0.13 | -0.24, -0.02 | 0.020 |  | 0.07 | -0.04, 0.19 | 0.212 |
| Year 2 | -0.21 | -0.30, -0.12 | <0.001 |  | -0.03 | -0.11, 0.05 | 0.490 |
| Year 3 | -0.27 | -0.43, -0.10 | 0.002 |  | -0.05 | -0.20, 0.09 | 0.470 |
| Year 4 | -0.13 | -0.24, -0.03 | 0.011 |  | 0.04 | -0.05, 0.13 | 0.405 |
| Year 5 | -0.15 | -0.26, -0.05 | 0.004 |  | 0.00 | -0.09, 0.10 | 0.985 |
| Year 6 | -0.14 | -0.23, -0.05 | 0.003 |  | 0.00 | -0.08, 0.08 | 0.967 |
| Year 7 | -0.17 | -0.28, -0.07 | 0.001 |  | 0.00 | -0.13, 0.12 | 0.947 |
| Year 8 | -0.30 | -0.43, -0.17 | <0.001 |  | -0.10 | -0.24, 0.05 | 0.205 |
| Year 9 | -0.18 | -0.45, 0.10 | 0.204 |  | -0.18 | -0.45, 0.10 | 0.204 |
| Year 10 | -0.46 | -0.75, -0.17 | 0.002 |  | 0.05 | -0.26, 0.35 | 0.769 |
| Year 11 | -0.83 | -1.21, -0.45 | <0.001 |  | -0.31 | -0.71, 0.10 | 0.134 |
| **(B) Small-for-gestational-age** (≤10% versus >10%) | | | | | | | |
| Authorised absences |  |  |  |  |  |  |  |
| Year 1 | 0.88 | 0.43, 1.33 | <0.001 |  | 0.37 | -0.07, 0.80 | 0.095 |
| Year 2 | 0.74 | 0.22, 1.25 | 0.005 |  | 0.26 | -0.24, 0.75 | 0.303 |
| Year 3 | 0.88 | 0.48, 1.27 | <0.001 |  | 0.51 | 0.11, 0.90 | 0.012 |
| Year 4 | 0.65 | 0.22, 1.09 | 0.003 |  | 0.38 | -0.07, 0.83 | 0.100 |
| Year 5 | 0.68 | 0.23, 1.14 | 0.003 |  | 0.41 | -0.05, 0.86 | 0.079 |
| Year 6 | 0.15 | -0.32, 0.63 | 0.529 |  | 0.00 | -0.49, 0.49 | 0.996 |
| Year 7 | 0.15 | -0.23, 0.54 | 0.432 |  | -0.07 | -0.43, 0.30 | 0.725 |
| Year 8 | 0.22 | -0.25, 0.68 | 0.360 |  | 0.04 | -0.41, 0.50 | 0.858 |
| Year 9 | -0.07 | -0.57, 0.43 | 0.780 |  | -0.07 | -0.57, 0.43 | 0.780 |
| Year 10 | 0.47 | -0.24, 1.19 | 0.189 |  | 0.25 | -0.46, 0.96 | 0.487 |
| Year 11 | -0.13 | -0.72, 0.47 | 0.672 |  | -0.27 | -0.89, 0.34 | 0.381 |
| Unauthorised absences |  |  |  |  |  |  |  |
| Year 1 | 0.27 | 0.07, 0.46 | 0.007 |  | 0.09 | -0.10, 0.28 | 0.347 |
| Year 2 | 0.39 | 0.18, 0.61 | <0.001 |  | 0.23 | 0.02, 0.45 | 0.033 |
| Year 3 | 0.58 | 0.13, 1.04 | 0.013 |  | 0.39 | -0.03, 0.81 | 0.066 |
| Year 4 | 0.20 | 0.00, 0.41 | 0.049 |  | 0.09 | -0.10, 0.27 | 0.376 |
| Year 5 | 0.21 | -0.02, 0.45 | 0.069 |  | 0.11 | -0.10, 0.32 | 0.313 |
| Year 6 | 0.18 | -0.01, 0.37 | 0.058 |  | 0.05 | -0.13, 0.23 | 0.563 |
| Year 7 | 0.46 | 0.13, 0.80 | 0.007 |  | 0.29 | -0.05, 0.64 | 0.096 |
| Year 8 | 0.63 | 0.13, 1.13 | 0.014 |  | 0.42 | -0.09, 0.93 | 0.102 |
| Year 9 | 0.64 | -0.07, 1.34 | 0.076 |  | 0.64 | -0.07, 1.34 | 0.076 |
| Year 10 | 0.52 | 0.07, 0.97 | 0.025 |  | 0.03 | -0.46, 0.52 | 0.905 |
| Year 11 | 1.38 | 0.38, 2.38 | 0.007 |  | 0.78 | -0.18, 1.74 | 0.110 |

Linear regressions with complex survey weights were fitted, models were adjusted for sex, month of birth, ethnic groups, partnership status, maternal age at birth, maternal smoking, number of siblings, household tenure, highest household education attainment, household income, and area-level income deprivation. n = 6275.

**Supplementary Table 14**: Adjusted associations between (a) birth weight, (b) small-for-gestational-age and key stage school attainments in non-preterm births (≥37 weeks).

|  | **Unadjusted** | | |  | **Adjusted** | | |
| --- | --- | --- | --- | --- | --- | --- | --- |
|  | b | 95% CI | *p* |  | b | 95% CI | *p* |
| **(A) Birth Weight** (per 1kg) | | | | | | | |
| Key Stage 1 |  |  |  |  |  |  |  |
| English (Reading) | 0.14 | 0.09, 0.20 | <0.001 |  | 0.09 | 0.04, 0.14 | <0.001 |
| Maths | 0.22 | 0.17, 0.27 | <0.001 |  | 0.13 | 0.09, 0.17 | <0.001 |
| Key Stage 2 |  |  |  |  |  |  |  |
| English | 0.17 | 0.12, 0.23 | <0.001 |  | 0.14 | 0.09, 0.19 | <0.001 |
| Maths | 0.23 | 0.18, 0.27 | <0.001 |  | 0.14 | 0.10, 0.18 | <0.001 |
| Key Stage 4 |  |  |  |  |  |  |  |
| English | 0.13 | 0.07, 0.18 | <0.001 |  | 0.11 | 0.06, 0.15 | <0.001 |
| Maths | 0.22 | 0.17, 0.27 | <0.001 |  | 0.13 | 0.09, 0.18 | <0.001 |
| Attainment 8 | 0.16 | 0.11, 0.21 | <0.001 |  | 0.11 | 0.07, 0.15 | <0.001 |
| **(B) Small-for-gestational-age** (≤10% versus >10%) | | | | | | | |
| Key Stage 1 |  |  |  |  |  |  |  |
| English (Reading) | -0.29 | -0.38, -0.19 | <0.001 |  | -0.19 | -0.27, -0.10 | <0.001 |
| Maths | -0.36 | -0.44, -0.28 | <0.001 |  | -0.25 | -0.31, -0.18 | <0.001 |
| Key Stage 2 |  |  |  |  |  |  |  |
| English | -0.30 | -0.42, -0.19 | <0.001 |  | -0.21 | -0.32, -0.10 | <0.001 |
| Maths | -0.27 | -0.36, -0.17 | <0.001 |  | -0.19 | -0.28, -0.10 | <0.001 |
| Key Stage 4 |  |  |  |  |  |  |  |
| English | -0.25 | -0.35, -0.14 | <0.001 |  | -0.16 | -0.26, -0.07 | 0.001 |
| Maths | -0.29 | -0.38, -0.19 | <0.001 |  | -0.19 | -0.28, -0.11 | <0.001 |
| Attainment 8 | -0.25 | -0.35, -0.16 | <0.001 |  | -0.17 | -0.25, -0.09 | <0.001 |

Linear regressions with complex survey weights were fitted, models were adjusted for sex, month of birth, ethnic groups, partnership status, maternal age at birth, maternal smoking, number of siblings, household tenure, highest household education attainment, household income, and area-level income deprivation. n = 5864.

**Supplementary Figure 8**: Associations between (a) birth weight, (b) small-for-gestational-age (compared to average-for-gestational-age) and persistent school absences (>10% of session missed). Linear regressions with complex survey weights were computed, associations were controlled for sex, month of birth, ethnic groups, partnership status, maternal age at birth, maternal smoking, number of siblings, household tenure, highest household education attainment, household income, and area-level income deprivation. n = 6659


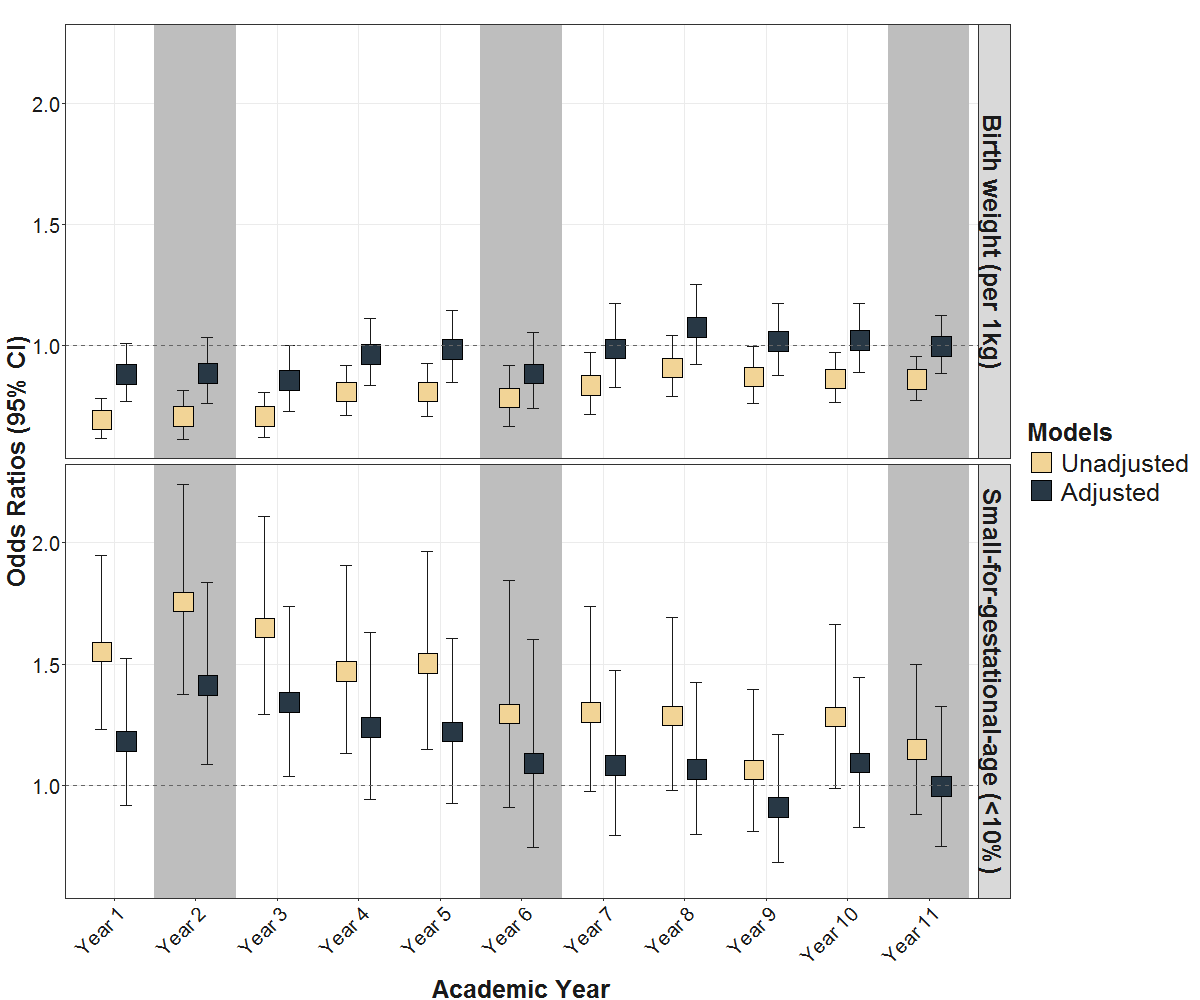

Supplement: online supplemental file 1 [file archdischild-110-6-s001.docx]
